# Active Metabolites From the Endophyte *Paenibacillus polymyxa* Y-1 of *Dendrobium nobile* for the Control of Rice Bacterial Diseases

**DOI:** 10.3389/fchem.2022.879724

**Published:** 2022-03-29

**Authors:** Wenshi Yi, Chao Chen, Xiuhai Gan

**Affiliations:** ^1^ State Key Laboratory Breeding Base of Green Pesticide and Agricultural Bioengineering, Key Laboratory of Green Pesticide and Agricultural Bioengineering, Ministry of Education, Guizhou University, Guiyang, China; ^2^ School of Chemistry and Materials Science, Guizhou Education University, Guiyang, China

**Keywords:** *Dendrobium nobile*, *Paenibacillus polymyxa* Y-1, active metabolites, rice bacterial diseases, antibacterial activity

## Abstract

Microbial bactericides have been a research hotspot in recent years. In order to find new microbial fungicides for preventing and treating rice bacterial diseases, *Paenibacillus polymyxa* Y-1 (*P. polymyxa* Y-1) was isolated from *Dendrobium nobile* in this study, and the optimal medium was selected by a single-factor experiment, and then eight metabolites were isolated from *P. polymyxa* Y-1 fermentation broth by bioactivity tracking separation. The bioassay results showed that 2,4-di-tert-butylphenol, N-acetyl-5-methoxytryptamine, and P-hydroxybenzoic acid have good antibacterial activity against *Xanthomonas oryzae* pv. *Oryzicola* (*Xoo*) and *Xanthomonas oryzae* pv. *oryzae* (*Xoc*), with 50% effective concentration values of 49.45 μg/ml, 64.22 μg/ml, and 16.32 μg/ml to *Xoo*, and 34.33 μg/ml, 71.17 μg/ml, and 15.58 μg/ml to *Xoc*, respectively, compared with zhongshengmycin (0.42 and 0.82 μg/ml, respectively) and bismerthiazol (85.64 and 92.49 μg/ml, respectively). *In vivo* experiments found that 2,4-di-tert-butylphenol (35.9 and 35.4%, respectively), N-acetyl-5-methoxytryptamine (42.9 and 36.7%, respectively), and P-hydroxybenzoic acid (40.6 and 36.8%, respectively) demonstrated excellent protective and curative activity against rice bacterial leaf blight, which were better than that of zhongshengmycin (38.4 and 34.4%, respectively). In addition, after 2,4-di-tert-butylphenol, N-acetyl-5-methoxytryptamine, and P-hydroxybenzoic acid acted on rice, SOD, POD, and CAD defense enzymes increased under the same condition. In conclusion, these results indicated that the activity and mechanism research of new microbial pesticides were helpful for the prevention and control of rice bacterial diseases.

## 1 Introduction

Plant bacterial diseases caused by microorganisms lead to tremendous economic losses to crops all over the world every year. Rice bacterial leaf blight (BLB) is caused by *Xanthomonas oryzae* pv. *oryzae* (*Xoo*) and results in reduced rice quality and yield with up to 40–80% loss (Cao et al., 2020; Zou et al., 2021). Traditional bactericides such as long-term use of bismerthiazol and thiodiazole copper will lead to drug resistance of pathogenic bacteria, which will affect the safety of the environment and plants, while microbial bactericides such as zhongshengmycin and shenqinmycin have low field control effects (Wang et al., 2019; Wang et al., 2021). Therefore, it is essential to discover new, highly active microbial antibacterial drugs.

*Dendrobium nobile* (*D. nobile*), a medicinal and edible plant, is native to China and belongs to the Orchidaceae family, which is rich in endophytic bacteria (Cai et al., 2015). Among them, *Paenibacillus polymyxa* (*P. polymyxa*) is an important endophytic bacterium from *D. nobile*, which can produce a variety of active metabolites, such as polymyxins (Niu et al., 2013; Mülner et al., 2021), paenibacillin (Huang and Yousef, 2015; Campbell et al., 2021), fusaricidins (Mikkola et al., 2017; Mülner et al., 2021), cytokinins (Liu et al., 2020), auxins (Sadhana and Tabacchioni, 2009), chitinase (Belén et al., 2020), and hydrolase(Sadhana and Tabacchioni, 2009). These metabolites can promote plant growth, improve plant nutrient utilization, and induce plant systemic resistance.

In previous work, three pairs of fusaricidin compounds were isolated from *P. polymyxa* Y-1. *In vitro* and *in vivo* activity studies found that fusaricidin compounds exhibited good antifungal activity against *Pestalotiopsis*. The mechanism study showed that fusaricidin compounds could inhibit amino acid biosynthesis and energy generation of *Pestalotiopsis* (Yang et al., 2018). In this study, the optimum nutrient medium was selected by a single factor experiment, and then eight metabolites were isolated from *P. polymyxa* Y-1 by bioactivity tracking separation. 2,4-Di-tert-butylphenol, N-acetyl-5-methoxytryptamine, and P-hydroxybenzoic acid exhibited good antibacterial activity to *Xoo* and *Xoc*. In addition, the *in vivo* activities of 2,4-di-tert-butylphenol, N-acetyl-5-methoxytryptamine, and P-hydroxybenzoic acid were investigated. It lays a foundation for the mechanism research of new microbial bactericides.

## 2 Materials and Methods

### 2.1 Samples, Strains, and Culture Condition

*D. nobile* samples were obtained from Chishui City, Guizhou Province, China. In previous work, *P. polymyxa* Y-1 has been isolated from *D. nobile* (Yang et al., 2018). *Xoo* and *Xoc* strains came from the State Key Laboratory Breeding Base of Green Pesticide and Agricultural Bioengineering, Ministry of Education, Guizhou University, China. *P. polymyxa* Y-1 was cultured at 28°C with nutrient broth (NB) medium and stored at –80°C in Luria-Bertani culture medium with 30% (v/v) glycerol. The *Xoo* and *Xoc* strains were cultured at 28°C with NB medium and stored at 4°C with nutrient agar medium.

### 2.2 Single-Factor Experiment

Single factors such as carbon source, nitrogen source, concentration, temperature, pH value, and time were selected to study their effects on the bacteriostatic ability of *P. polymyxa* Y-1 fermentation broth. Using 10 g/L peptone, 0.4 g/L MgSO_4_ and 2 g/L KH_2_PO_4_ as basal medium (PH = 7), 30 g/L lactose, glucose, maltose, fructose, glycerol, and starch carbon sources were respectively added to prepare different carbon sources culture medium (200 ml), and then *P. polymyxa* Y-1 strain (10 ml) was inoculated. Using 20 g/L glycerol, 0.4 g/L MgSO_4_, and 2 g/L KH_2_PO_4_ as basal medium (PH = 7), 10 g/L peptone, tryptone, beef extract, yeast powder, and urea nitrogen sources were respectively added to prepare different nitrogen sources culture medium (200 ml), and then *P. polymyxa* Y-1 strain (10 ml) was inoculated. Using 0.4 g/L MgSO_4_ and 2 g/L KH_2_PO_4_ as the basal medium (PH = 7), carbon source and nitrogen source were respectively added to prepare concentrations of 50, 40, 30, 20, and 10 g/L nutrient medium (200 ml), and then *P. polymyxa* Y-1 strain (10 ml) was inoculated. The effect of pH value showed the pH value was adjusted to 5.0, 5.5, 6.0, 6.5, 7.0, 7.5, and 8.0 with 1 mol/L HCL and NaOH in the basal medium (200 ml), and then *P. polymyxa* Y-1 strain (10 ml) was inoculated. The temperature effect showed that *P. polymyxa* Y-1 was cultured on a shaking table at 25, 26, 27, 28, 29, and 30°C, respectively. The time effect showed that *P. polymyxa* Y-1 was incubated on a shaker for 48, 72, 96, 120, 144, 168, 192, and 216 h, respectively. The paper disk method was used to detect the antibacterial activity of the *P. polymyxa* Y-1 fermentation broth to *Xoo* and *Xoc*. The experiments were all performed three times and in triplicate (Miao et al., 2012).

### 2.3 Antibacterial Activity Experiments

The paper disk method was used to detect the antibacterial activity of the *P. polymyxa* Y-1 fermentation broth to *Xoo* and *Xoc.* The turbidimetric method was used to evaluate the *in vitro* antibacterial activities of the metabolites from *P. polymyxa* Y-1 to *Xoo* or *Xoc* (Dalgaard et al., 1994; Happy, 2017). The protective and curative activities of active metabolites against BLB were measured in potted rice by Schaad’s method (Li et al., 2018). Zhongshengmycin (12% wettable powder) was used as positive controls. SPSS 17.0 software was used to calculate 50% effective concentration (EC_50_) values of the active metabolites. These experiments were all performed three times and in triplicate.

### 2.4 Extraction, Isolation, and Structural Identification of Metabolites From *P. polymyxa* Y-1

**FIGURE 1:**
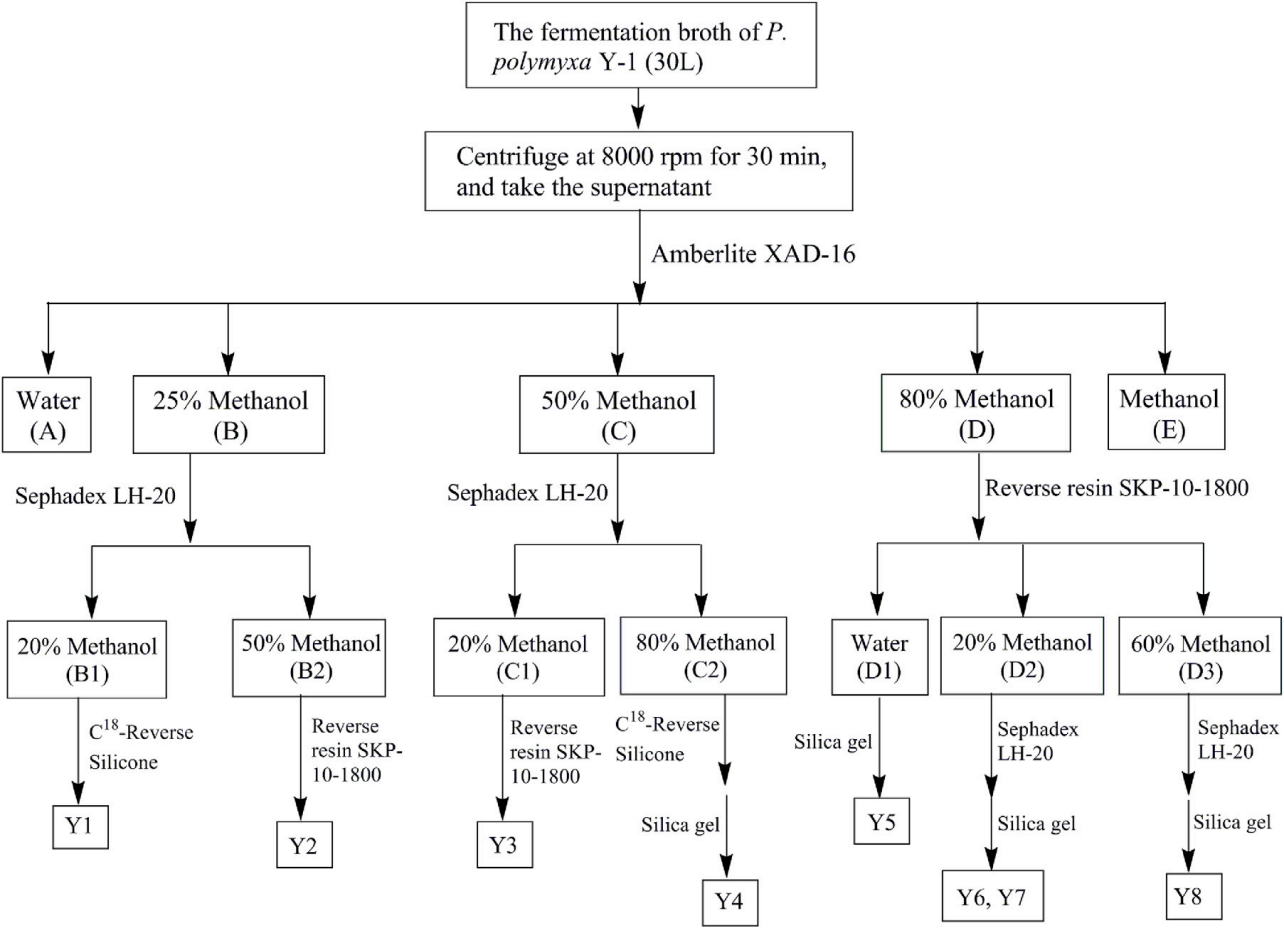
Metabolites extraction, separation, and purification flow chart of *P. polymyxa* Y-1.

The metabolites were separated from the *P. Polymyxa* Y-1 fermentation broth by biological activity tracking separation, the separation process is shown in Figure 1. The fermentation broth was centrifuged to take the supernatant, which was placed on an Amberlite XAD-16 column and eluted with water–methanol, the eluant polarity was gradually reduced, the ratios of pure water to methanol were 100:0 (A), 75:25 (B), 50:50 (C), 20:80 (D), and 0:100 (E) in turn. The same polarity sections were combined and concentrated into a yellow solid. The solid of B (18.3 g) segment was redissolved in 1 L pure water, filtered through a 0.22 μm microporous membrane filter, and loaded onto a Sephadex LH-20 column. The column was eluted with 20% (B1), and 50% (B2) methanol in turn at a rate of 6 s per drop. Approximately, 50 ml from each of the eluant was collected, and the same segments were enriched and concentrated. The B1 (292.4 mg) sub-fractions was placed on a Phenomenex Gemini C^18^ (00G-4435-N0) and eluted with 0.1% trifluoroacetic acid (TFA) water–acetonitrile to obtain Y1 (32.5 mg). The B2 (167.8 mg) sub-fractions was placed in SKP-10-1800 reverse-phase resin and eluted with methanol to obtain Y2 (24.7 mg). The solid of C (876 mg) segment was redissolved in pure water (600 ml), filtered using a 0.22 μm microporous membrane filter, and loaded onto a Sephadex LH-20 column. The column was eluted with 20% (C1) and 80% (C2) methanol in turn at a rate of 6 s per drop. The C1 (187.4 mg) sub-fractions were placed in SKP-10-1800 reverse-phase resin and eluted with water–methanol to obtain Y3 (40.5 mg). The C2 (123.4 mg) sub-fractions were placed on a Phenomenex Gemini C^18^ (00G-4435-N0) and eluted with 0.1% TFA water–acetonitrile to obtain Y4 (26.7 mg). The solid of D (10.2 g) segment was redissolved in 800 ml pure water, filtered using a 0.22 μm microporous membrane filter, and loaded onto a SKP-10-1800 reverse-phase resin. The column was eluted with water (D1), 20% (D2) and 60% (D3) methanol in turn at a rate of 6 s per drop. The D1 (198.5 mg) sub-fractions was placed in a silica gel column and eluted with dichloromethane–methanol to obtain Y5 (26.3 mg). The D2 (273.4 mg) and D3 (123.8 mg) sub-fractions were respectively placed on a Sephadex LH-20 column and eluted with water–methanol to obtain Y6 (18.2 mg), Y7 (32.9 mg), and Y8 (16.5 mg). The structures were identified by using ^1^H NMR, ^13^C NMR and high resolution mass spectrometry.

### 2.5 Defensive Enzyme Activity Detection

The protective activities of active metabolites against BLB were determined in potted rice by Schaad’s method, and then the samples were harvested on the 1st, 3rd, 5th, and 7th days. The activities of superoxide dismutase (SOD), peroxidase (POD), and cinnamyl alcohol dehydrogenase (CAD) were measured, according to the instructions of the enzyme assay reagent kits (Beijing Solarbao Life Sciences, China). These experiments were all performed three times and in triplicate.

## 3 Results and Discussion

### 3.1 Antibacterial Activity Assay of *P. polymyxa* Y-1 Supernatant

The *in vitro* antibacterial activity of the *P. polymyxa* Y-1 fermentation broth against *Xoo* and *Xoc* was determined by the paper disk method, and the bioassay results showed that the bacteriostatic rates of *P. polymyxa *Y-1 fermentation broth against *Xoo* and *Xoc* were 34.29 and 30.16%, respectively (Supplementary Figure S1).

### 3.2 Single Factor Experiment Assay

The bacteriostatic rates of *P. polymyxa* Y-1 fermentation broth under different culture conditions were maintained to determine the optimal culture conditions.

#### 3.2.1 Effects of Different Carbon Sources on the Resistance of *P. polymyxa* Y-1

**FIGURE 2:**
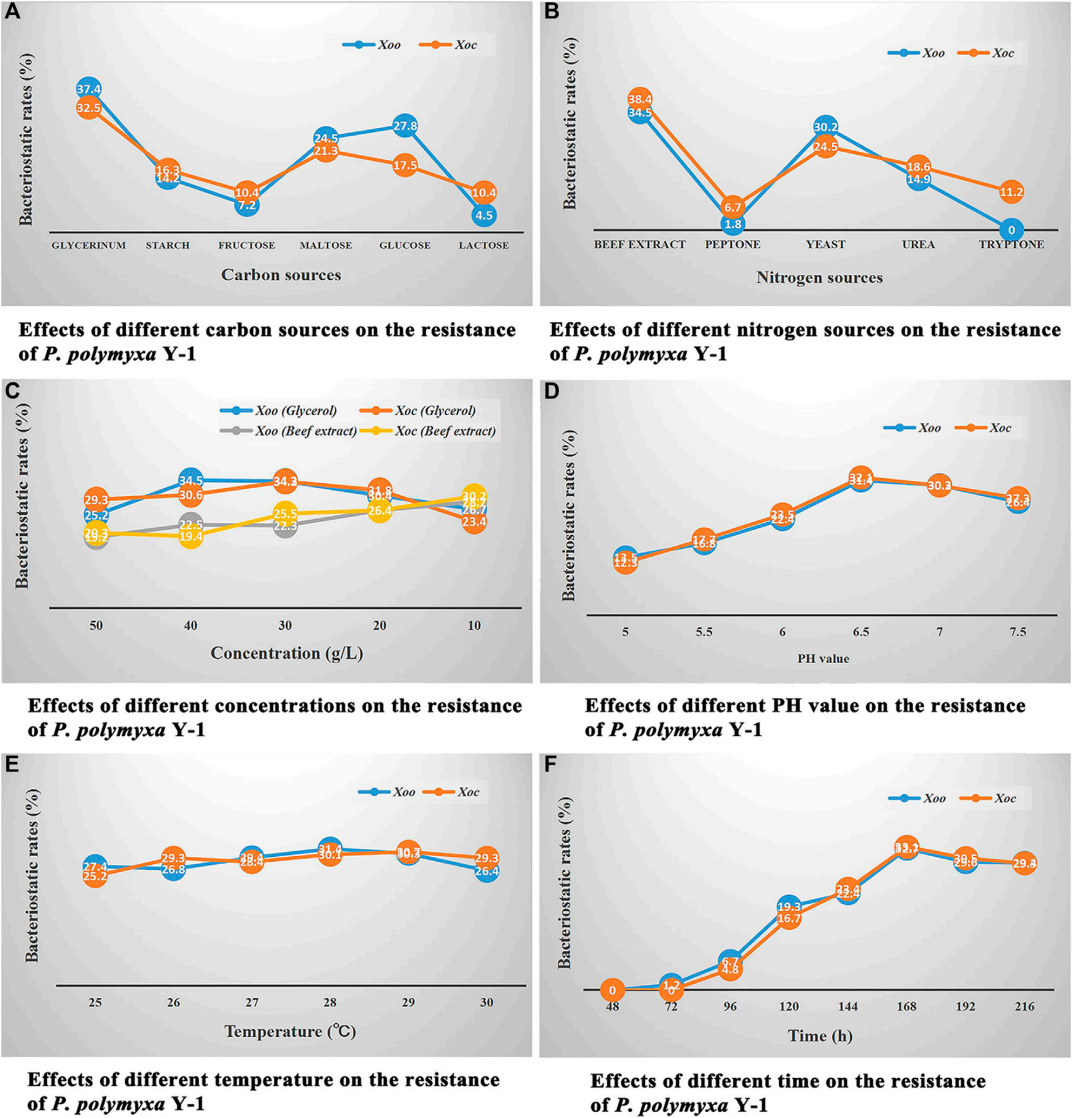
Single factor experiment assay. **(A)** Effects of different carbon sources on the resistance of *P. polymyxa* Y-1. **(B)** Effects of different nitrogen sources on the resistance of *P. polymyxa* Y-1. **(C)** Effects of different concentrations on the resistance of *P. polymyxa* Y-1. **(D)** Effects of different pH on the resistance of *P. polymyxa* Y-1. **(E)** Effects of different temperature on the resistance of *P. polymyxa* Y-1. **(F)** Effects of different time on the resistance of *P. polymyxa* Y-1.

As shown in Figure 2A, when glycerol, starch, fructose, maltose, glucose, and lactose were the carbon sources of the nutrient medium, different nutrient medium were used to culture *P. polymyxa* Y-1, the *in vitro* bacteriostatic rates of its fermentation broth against *Xoo* were 37.4, 14.2, 7.2, 24.5, 27.8, and 4.5%, respectively, the *in vitro* bacteriostatic rates against *Xoc* were 32.5, 16.3, 10.4, 21.3, 17.5, and 10.4%, respectively. The results showed that glycerol was the best carbon source, and the *in vitro* bacteriostatic rates of the *P. polymyxa* Y-1 fermentation broth to *Xoo* and *Xoc* were 37.4 and 32.5%, respectively.

#### 3.2.2 Effects of Different Nitrogen Sources on the Resistance of *P. polymyxa* Y-1

As shown in Figure 2B, when beef extract, peptone, yeast, urea, and tryptone were the nitrogen sources of the nutrient medium, different nutrient medium were used to culture *P. polymyxa* Y-1, the *in vitro* bacteriostatic rates of its fermentation broth against *Xoo* were 34.5, 1.8, 30.2, 14.9, 0%, respectively, the *in vitro* bacteriostatic rates against *Xoc* were 38.4, 6.7, 24.5, 18.6, 11.2%, respectively. The results showed that the beef extract was the best nitrogen source, the *in vitro* bacteriostatic rates of the *P. polymyxa* Y-1 fermentation broth to *Xoo* and *Xoc* were 34.5 and 38.4%, respectively.

#### 3.2.3 Effects of Different Concentrations on the Resistance of *P. polymyxa* Y-1

As shown in Figure 2C, when the concentration of the beef extract was fixed and the concentration of glycerol was 50, 40, 30, 20, and 10 g/L, respectively, the nutrient medium with different carbon sources were used to culture *P. polymyxa* Y-1, the *in vitro* bacteriostatic rates of its fermentation broth against *Xoo* were 25.2, 34.5, 34.3, 30.4, and 26.7%, respectively, the *in vitro* bacteriostatic rates against *Xoc* were 29.3, 30.6, 34.2, 31.8, and 23.4%, respectively. When the concentration of glycerol was fixed and the concentration of the beef extract were 50, 40, 30, 20, and 10 g/L, respectively, the nutrient medium with different nitrogen sources were used to culture *P. polymyxa* Y-1, the *in vitro* bacteriostatic rates of its fermentation broth against *Xoo* were 19.2, 22.5, 22.3, 26.4, and 28.7%, respectively, the *in vitro* bacteriostatic rates against *Xoc* were 20.3, 19.4, 25.5, 26.4, and 30.2%, respectively. The results showed that the optimal concentration of glycerol was 30 g/L, and the *in vitro* bacteriostatic rates of *P. polymyxa* Y-1 fermentation broth against *Xoo* and *Xoc* were 34.3 and 34.2%, respectively. The optimal concentration of the beef extract was 10 g/L, and the *in vitro* bacteriostatic rates of *P. polymyxa* Y-1 fermentation broth against *Xoo* and *Xoc* were 28.7 and 30.2%, respectively.

#### 3.2.4 Effects of Different pH Values on the Resistance of *P. polymyxa* Y-1

As shown in Figure 2D, when the pH values were adjusted to 5.0, 5.5, 6.0, 6.5, 7.0, and 7.5, respectively, different nutrient medium were used to culture *P. polymyxa* Y-1, the *in vitro* bacteriostatic rates of its fermentation broth against *Xoo* were 13.5, 16.8, 22.4, 31.4, 30.3, and 26.4%, respectively, the *in vitro* bacteriostatic rates against *Xoc* were 12.3, 17.7, 23.5, 32.1, 30.2, and 27.3%, respectively. The results showed that the optimum pH value was 6.5, and the *in vitro* bacteriostatic rates of the *P. polymyxa* Y-1 fermentation broth against *Xoo* and *Xoc* were 31.4 and 32.1%, respectively.

#### 3.2.5 Effects of Different Temperatures on the Resistance of *P. polymyxa* Y-1

As shown in Figure 2E, when the culture temperature were 25, 26, 27, 28, 29, and 30°C, respectively, the *in vitro* bacteriostatic rates of *P. polymyxa* Y-1 fermentation broth against *Xoo* were 27.4, 26.8, 29.4, 31.4, 30.3, and 26.4%, respectively, the *in vitro* bacteriostatic rates against *Xoc* were 25.2, 29.3, 28.4, 30.1, 30.7, and 29.3%, respectively. The results showed that the optimum temperature was 28°C, and the *in vitro* bacteriostatic rates of the *P. polymyxa* Y-1 fermentation broth against *Xoo* and *Xoc* were 31.4 and 30.1%, respectively.

#### 3.2.6 Effects of Different Time on the Resistance of *P. polymyxa* Y-1

As shown in Figure 2F, When the culture time were 48, 72, 96, 120, 144, 168, 192, and 216 h, respectively, the *in vitro* bacteriostatic rates of *P. polymyxa* Y-1 fermentation broth against *Xoo* were 0, 1.2, 6.7, 19.3, 22.4, 32.7, 29.6, and 29.4%, respectively, the *in vitro* bacteriostatic rates against *Xoc* were 0, 0, 4.8, 16.7, 23.4, 33.1, 30.5, and 29.3%, respectively. The results showed that the optimum time was 168 h, and the *in vitro* bacteriostatic rates of the *P. polymyxa* Y-1 fermentation broth against *Xoo* and *Xoc* were 32.7 and 33.1%, respectively.

### 3.3 Structure Identification of Metabolites

**FIGURE 3:**
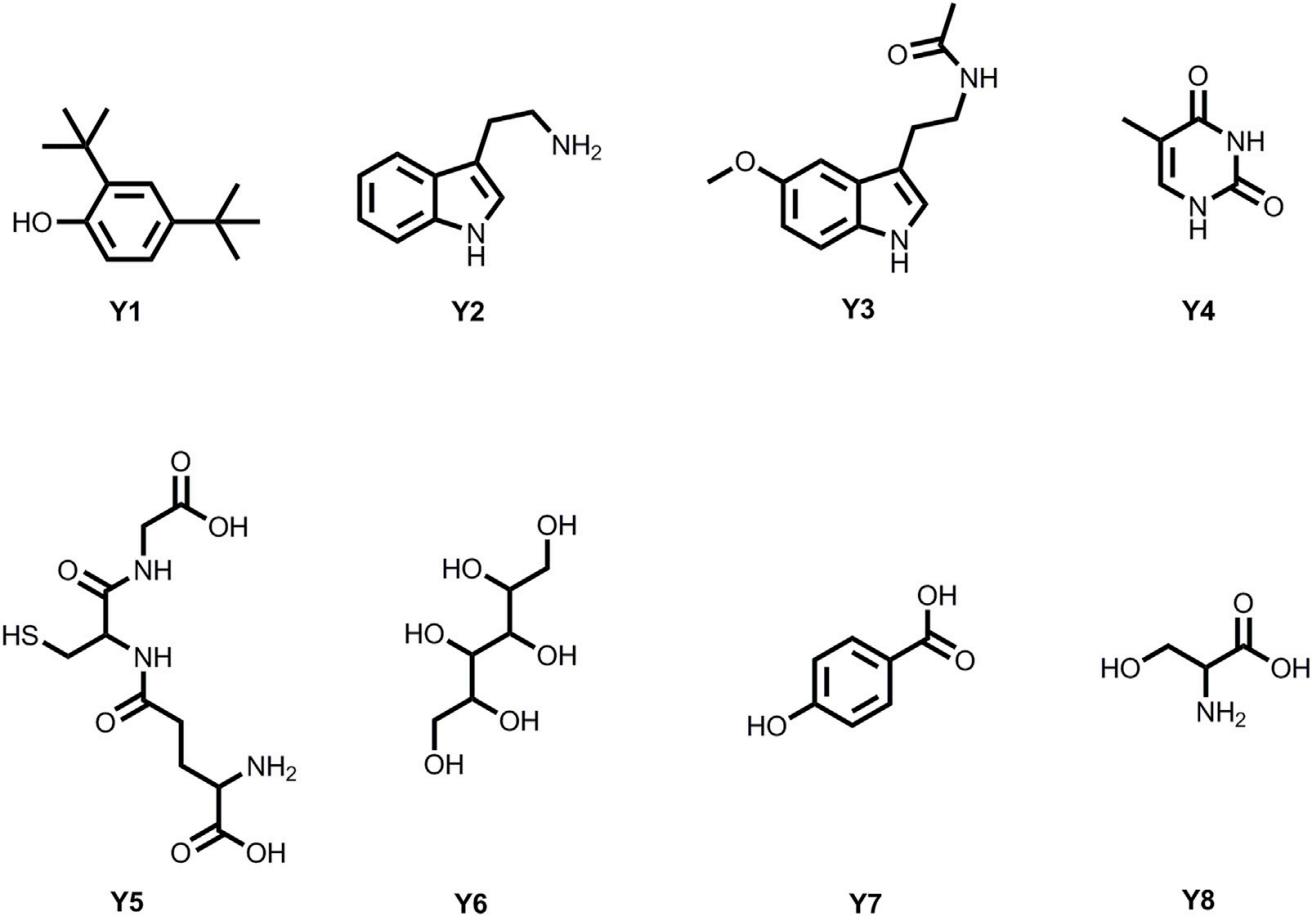
Chemical structures of eight metabolites.

Eight metabolites were isolated from *P. polymyxa* Y-1 fermentation broth with bioactivity tracking separation (Figure 3); the structures of the metabolites were confirmed by using ^1^H NMR, ^13^C NMR, and HRMS data (Supplementary Material).

#### 3.3.1 2,4-Di-tert-butylphenol (Y1)

Light yellow solid; m.p. 53–56°C. ^1^H NMR (500 MHz, CD_3_OD) δ 7.17 (d, *J* = 2.44 Hz, 1H), 6.94 (dd, *J* = 8.30, 2.50°Hz, 1H), 6.58 (d, *J* = 8.29°Hz, 1H), 1.34 (s, 9H), 1.21 (s, 9H); ^13^C NMR (125 MHz, CD_3_OD) δ 153.4, 140.9, 134.8, 123.0, 122.9, 115.3, 34.4, 33.7, 31.0, 28.9. HRMS (ESI): calculated for C_14_H_21_O [M-H]^-^: 205.15979, found: 205.16041 (Seema et al., 2014).

#### 3.3.2 3-(2-Aminoethyl) Indole (Y2)

Light yellow powder; m.p. 113–116°C. ^1^H NMR (400 MHz, DMSO-*d*_6_) δ 10.81 (s, 1H), 7.52 (d, *J* = 7.83°Hz, 1H), 7.34 (d, *J* = 8.07°Hz, 1H), 7.13 (d, *J* = 1.83°Hz, 1H), 7.08–7.04 (m, 1H), 6.99–6.95 (m, 1H), 2.84–2.74 (m, 4H), 1.57 (s, 2H). ^13^C NMR (101 MHz, DMSO-*d*_6_) δ 136.7, 127.8, 123.0, 121.3, 118.8, 118.6, 113.1, 111.8, 43.2, 30.1. HRMS (ESI): calculated for C_10_H_11_N_2_ [M-H]^-^: 159.09277, found: 159.09279 (Güngőr et al., 1994).

#### 3.3.3 N-Acetyl-5-Methoxytryptamine (Y3)

White crystal powder; m.p. 116–118°C. ^1^H NMR (400 MHz, DMSO-*d*_6_) δ 10.64 (s, 1H), 7.94 (t, *J* = 5.24°Hz, 1H), 7.23 (d, *J* = 8.73°Hz, 1H), 7.10 (d, *J* = 2.17°Hz, 1H), 7.02 (d, *J* = 2.34°Hz, 1H), 6.72 (dd, *J* = 8.73, 2.40°Hz, 1H), 3.76 (s, 3H), 3.31 (dd, *J* = 16.00, 8.00°Hz, 2H), 2.78 (t, *J* = 7.41°Hz, 2H), 1.81 (s, 3H); ^13^C NMR (101 MHz, DMSO-*d*_6_) δ 169.5, 153.4, 131.8, 128.0, 123.7, 112.4, 112.2, 111.5, 100.6, 55.8, 39.9, 25.7, 23.2. HRMS (ESI): calculated for C_13_H_15_N_2_O_2_ [M-H]^-^: 231.11390, found: 231.11430 (Hwang and Lee, 1999).

#### 3.3.4 2,4-Dihydroxy-5-Methylpyrimidine (Y4)

Light yellow powder; m.p. 316–317°C. ^1^H NMR (500 MHz, DMSO-*d*_6_) δ 11.02 (s, 1H), 10.60 (s, 1H), 7.25 (s, 1H), 1.72 (d, *J* = 1.1°Hz, 3H). ^13^C NMR (125 MHz, DMSO-*d*_6_) δ 165.5, 152.0, 138.3, 108.2, 12.3. HRMS (ESI): calculated for C_5_H_7_N_2_O_2_ [M + H]^+^: 127.05020, found: 127.01517 (Cadet et al., 1975).

#### 3.3.5 Glutathione (Y5)

Yellow powder; m.p. 192–195°C. ^1^H NMR (400 MHz, D_2_O**)** δ 4.60 (t, *J* = 6.09°Hz, 1H), 4.00 (s, 2H), 3.86 (t, *J* = 6.37°Hz, 1H), 3.02–2.92 (m, 2H),2.61–2.56 (m, 2H), 2.20 (dd, *J* = 16.00, 8.00°Hz, 2H); ^13^C NMR (101 MHz, D_2_O**)** δ 174.9, 173.6, 173.6, 172.4, 55.6, 53.8, 41.6, 31.2, 26.0, 25.4. HRMS (ESI): calculated for C_10_H_16_N_3_O_6_S [M-H]^−^: 306.07653, found: 306.07667 (Fujiwara et al., 1977).

#### 3.3.6 Mannitol (Y6)

White crystal powder; m.p. 167–170°C. ^1^H NMR (400 MHz, DMSO-*d*_6_) δ 4.41 (d, *J* = 5.48°Hz, 2H), 4.32 (t, *J* = 5.69°Hz, 2H), 4.13 (d, *J* = 7.05°Hz, 2H), 3.64–3.59 (m, 2H), 3.55 (t, *J* = 7.50°Hz, 2H), 3.49–3.43 (m, 2H), 3 41–3.35 (m, 2H); ^13^C NMR (101 MHz, DMSO-*d*_6_) δ 71.8, 70.1, 64.3. HRMS (ESI): calculated for C_6_H_13_O_6_ [M-H]^-^: 181.07176, found: 181.07159 (Tarczynski et al., 1992).

#### 3.3.7 P-Hydroxybenzoic Acid (Y7)

Light yellow crystal; m.p. 213–215°C. ^1^H NMR (400 MHz, CD_3_OD**)** δ 7.84 (d, *J* = 8.62°Hz, 2H) 6.78 (d, *J* = 8.72°Hz, 2H). ^13^C NMR (101 MHz, CD_3_OD) δ 168.7, 162.0, 131.6, 121.3, 114.7. HRMS (ESI): calculated for C_7_H_5_O_3_ [M-H]^−^: 137.02442, found: 137.02449 (Hsieha et al., 2005).

#### 3.3.8 Serine (Y8)

White powder; m.p. 239–241°C. ^1^H NMR (400 MHz, D_2_O) δ 4.06–3.96 (m, 2H) 3.91–3.88 (m, 1H). ^13^C NMR (101 MHz, D_2_O) δ 172.4, 60.2, 55.4. HRMS (ESI): calculated for C_3_H_6_NO_3_ [M-H]^-^: 104.03532, found: 104.03470 (Pogliani and Ziessow, 1981).

### 3.4 *In vitro* Antibacterial Activity Assays of Metabolites

The *in vitro* bacteriostatic rates of the eight metabolites and two positive control drugs against *Xoo* and *Xoc* were determined by the turbidimeter tests, and the results are shown in Table 1. The result showed that when the concentrations of the metabolites were 200 and 100 *μ*g/ml, 2,4-di-tert-butylphenol (82.57 and 72.31%, 80.14 and 75.24%, respectively), N-acetyl-5-methoxytryptamine (51.81 and 32.43%, 50.12 and 40.37%, respectively) and P-hydroxybenzoic acid (96.72 and 95.46%, 98.13 and 96.54%, respectively) exhibited excellent *in vitro* antibacterial activities to *Xoo* and *Xoc*, which was better than bismerthiazol (76.48 and 60.21%, 79.24 and 59.16%, respectively) and lower than zhongshengmycin (100%).

**TABLE 1:** *In vitro* antibacterial activity of metabolites and positive control drugs against *Xoo* and *Xoc*.

Serial number	Metabolite	*Xoo* (Ininbition rate %)		*Xoc* (Ininbition rate %)	
		100 *μ*g/ml	200 *μ*g/ml	100 *μ*g/ml	200 *μ*g/ml
1	2,4-di-tert-butylphenol (Y1)	72.31 ± 4.06	82.57 ± 2.45	75.24 ± 3.17	80.14 ± 1.29
2	3-(2-aminoethyl) indole (Y2)	12.17 ± 1.26	22.54 ± 4.23	15.62 ± 2.67	27.45 ± 1.74
3	N-acetyl-5-methoxytryptamine (Y3)	32.43 ± 4.25	51.81 ± 2.30	40.37 ± 6.12	50.12 ± 3.44
4	2,4-dihydroxy-5- methylpyrimidin (Y4)	4.81 ± 0.51	16.54 ± 1.94	3.24 ± 0.22	8.42 ± 0.70
5	Glutathione (Y5)	21.37 ± 0.22	35.42 ± 1.61	12.35 ± 0.44	32.41 ± 1.12
6	Mannitol (Y6)	5.41 ± 0.73	6.22 ± 0.91	4.92 ± 0.50	5.47 ± 0.60
7	P-hydroxybenzoic acid (Y7)	95.46 ± 5.32	96.72 ± 3.81	96.54 ± 4.47	98.13 ± 3.53
8	Serine (Y8)	22.16 ± 1.91	32.23 ± 4.26	12.11 ± 2.40	22.54 ± 1.43
9	Zhongshengmycin	100 ± 5.21	100 ± 3.18	100 ± 2.37	100 ± 3.24
10	Bismerthiazol	60.21 ± 2.78	76.48 ± 1.64	59.16 ± 3.72	79.24 ± 2.53

The EC_50_ values of 2,4-di-tert-butylphenol, P-hydroxybenzoic acid, and N-acetyl-5-methoxytryptamine are shown in Table 2. 2,4-di-tert-butylphenol, N-acetyl-5-methoxytryptamine, and P-hydroxybenzoic acid exhibited good antibacterial activity to *Xoo*, with EC_50_ values of 49.45 μg/ml, 64.22 μg/ml, and 16.32 μg/ml, respectively, which were better than bismerthiazol (85.64 μg/ml) and lower than zhongshengmycin (0.42 μg/ml). Meanwhile, 2,4-di-tert-butylphenol, N-acetyl-5-methoxytryptamine, and P-hydroxybenzoic acid also showed good antibacterial activity to *Xoc*, with EC_50_ values of 34.33 μg/ml, 71.17 μg/ml, and 15.58 μg/ml, respectively, which were better than bismerthiazol (92.49 μg/ml) and lower than zhongshengmycin (0.82 μg/ml). The results showed that 2,4-di-tert-butylphenol, N-acetyl-5-methoxytryptamine, and P-hydroxybenzoic acid had better bacteriostatic rates and could be used as new antibacterial agents.

**TABLE 2:** EC_50_ values of the 2,4-di-tert-butylphenol, N-acetyl-5-methoxytryptamine, P-hydroxybenzoic acid, zhongshengmycin, and bismerthiazol against *Xoo* and *Xoc*.

Serial number	Metabolite	*Xoo* EC_50_ (*μ*g/ml)	*Xoc* EC_50_ (*μ*g/ml)
1	2,4-di-tert-butylphenol (Y1)	49.45 ± 0.07	34.33 ± 0.12
2	N-acetyl-5-methoxytryptamine (Y3)	64.22 ± 2.16	71.17 ± 1.37
3	P-hydroxybenzoic acid (Y7)	16.32 ± 0.04	15.58 ± 0.06
4	Zhongshengmycin	0.42 ± 0.08	0.82 ± 0.05
5	Bismerthiazol	85.64 ± 3.24	92.49 ± 3.72

### 3.5 *In vivo* Antibacterial Activity Assay

**FIGURE 4:**
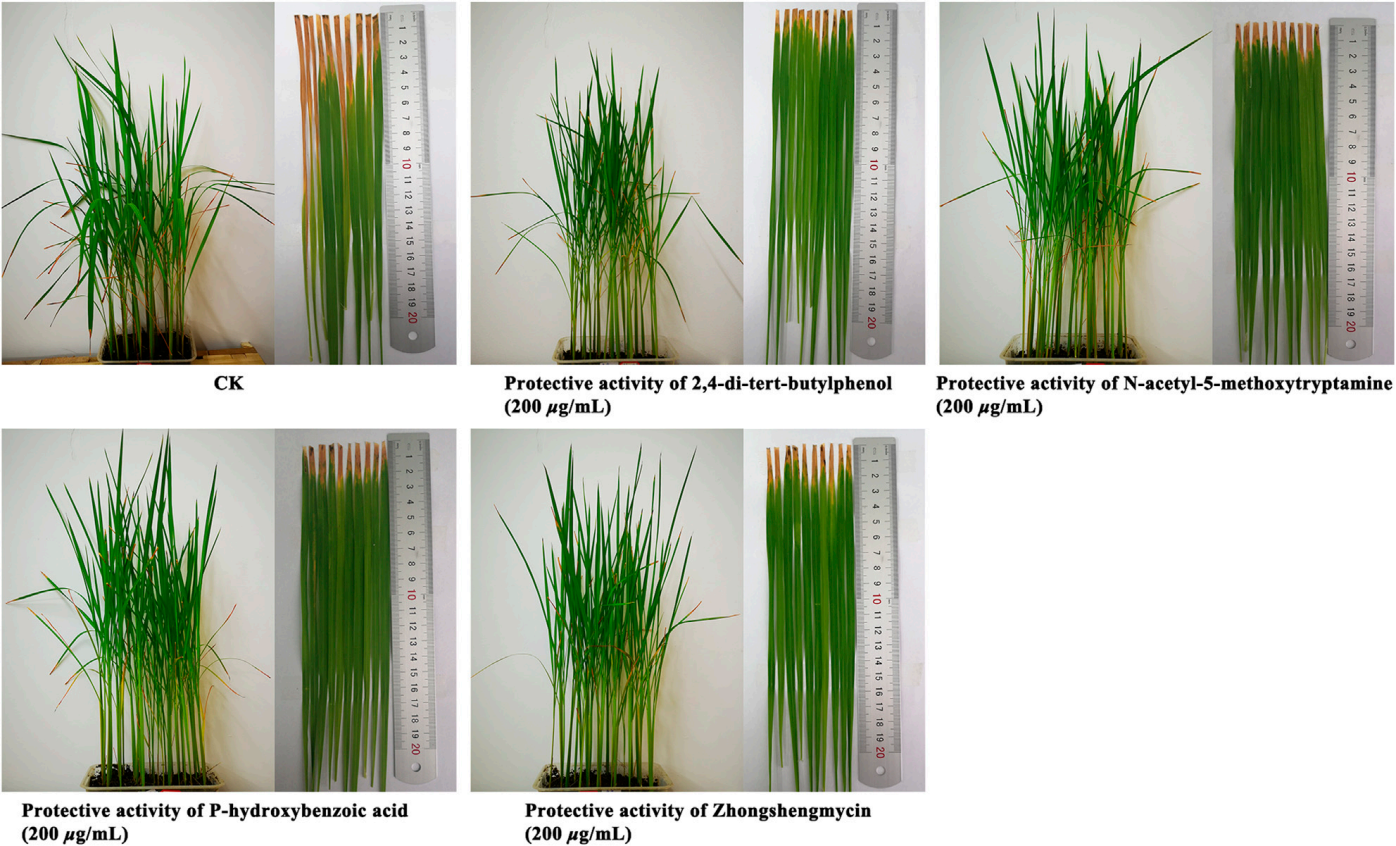
Protective activity of 2,4-di-tert-butylphenol, N-acetyl-5-methoxytryptamine, P-hydroxybenzoic acid against BLB at concentrations of 200 μg/ml.

**FIGURE 5:**
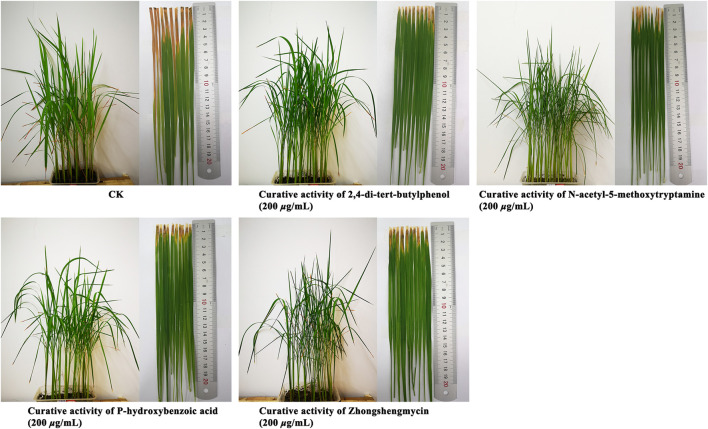
Curative activity of 2,4-di-tert-butylphenol, N-acetyl-5-methoxytryptamine, P-hydroxybenzoic acid against BLB at concentrations of 200 μg/ml.

The *In vivo* antibacterial activities of 2,4-di-tert-butylphenol, N-acetyl-5-methoxytryptamine, and P-hydroxybenzoic acid are shown in Table 3 and Figure 4. Under the greenhouse conditions, when the concentration was 200 μg/ml, the protective activity of 2,4-di-tert-butylphenol (35.9%) was lower than that of positive control drugs, zhongshengmycin (38.4%), the protective activity of N-acetyl-5-methoxytryptamine (42.9%) and P-hydroxybenzoic acid (40.6%) against BLB were better than that of zhongshengmycin (38.4%). As shown in Table 3 and Figure 5, when the concentration was 200 μg/ml, the curtive activity of 2,4-di-tert-butylphenol (35.4%), N-acetyl-5-methoxytryptamine (36.7%), and P-hydroxybenzoic acid (36.8%) against BLB were similar to that of zhongshengmycin (34.4%). The results indicated that 2,4-di-tert-butylphenol, N-acetyl-5-methoxytryptamine, and P-hydroxybenzoic acid significantly reduced the occurrence of BLB disease.

**TABLE 3:** Protective activity of 2,4-di-tert-butylphenol, N-acetyl-5-methoxytryptamine, P-hydroxybenzoic acid, and zhongshengmycin against BLB at different concentrations (greenhouse conditions).

Treatment	Protection activity (200 *μ*g/ml)		Curative activity (200 *μ*g/ml)	
	Infection index (%)	Control efficiency (%)	Infection index (%)	Control efficiency (%)
2,4-di-tert-butylphenol (Y1)	50.2 ± 2.1	35.9 ± 1.6	50.6 ± 3.4	35.4 ± 2.7
N-acetyl-5-methoxytryptamine (Y3)	44.7 ± 1.5	42.9 ± 1.7	49.6 ± 1.4	36.7 ± 1.7
P-hydroxybenzoic acid (Y7)	46.5 ± 2.7	40.6 ± 1.6	49.5 ± 2.5	36.8 ± 1.9
Zhongshengmycin	48.2 ± 1.2	38.4 ± 1.7	51.4 ± 2.1	34.4 ± 2.4
CK	78.3 ± 1.4	/	78.3 ± 1.4	/

### 3.6 Defensive Enzyme Activities

**FIGURE 6:**
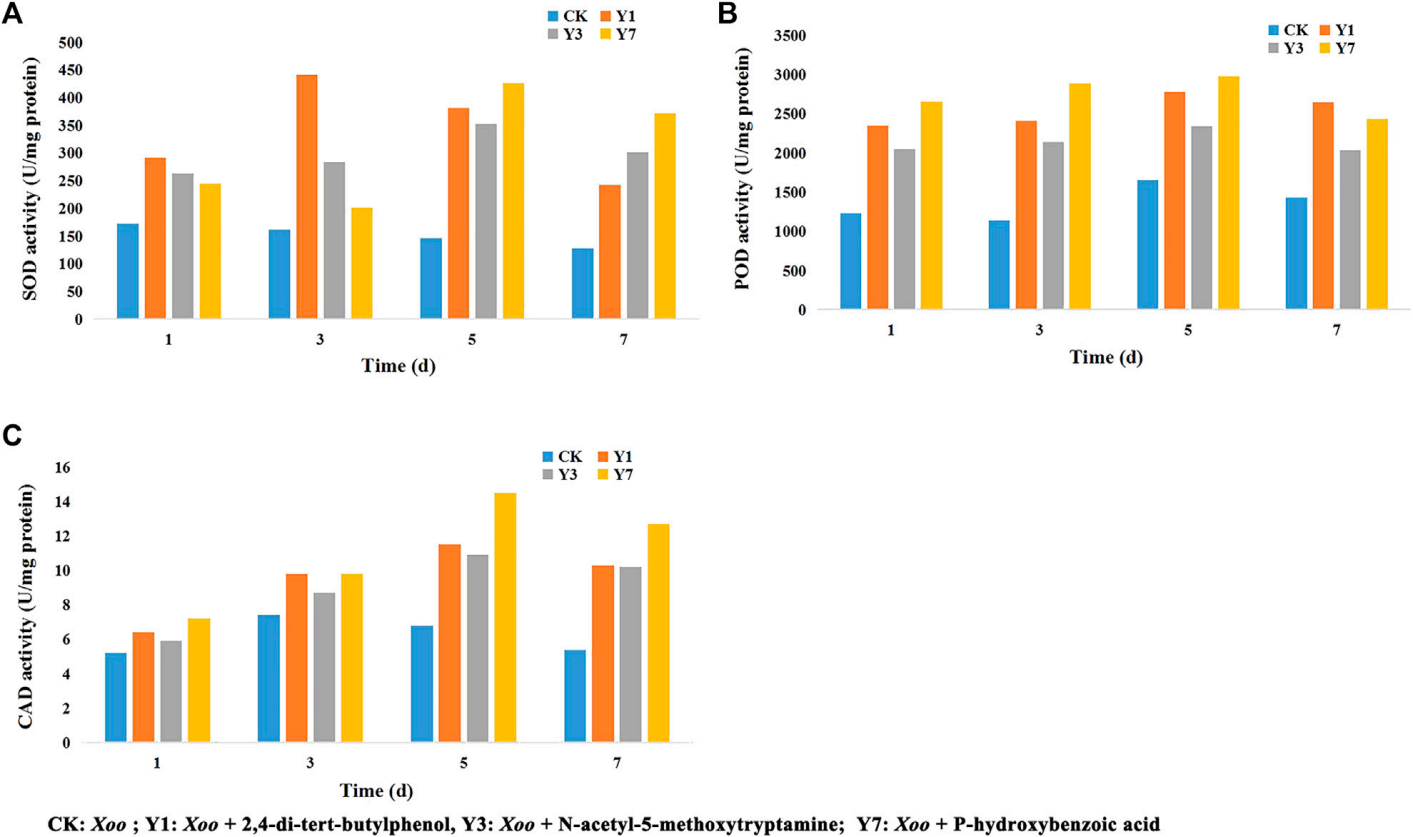
Effects of 2,4-di-tert-butylphenol, N-acetyl-5-methoxytryptamine, and P-hydroxybenzoic acid on SOD **(A)**, POD **(B)**, and CAD **(C)** activities in rice leaves.

As shown in Figure 6, the CK group was inoculated with *Xoo* after Tween water protection, the Y1 group was inoculated with *Xoo* after 2,4-di-tert-butylphenol protection, the Y3 group was inoculated with *Xoo* after N-acetyl-5-methoxytryptamine protection, and the Y7 group was inoculated with *Xoo* after P-hydroxybenzoic acid protection. The SOD activity was significantly increased in the Y1 treatment group and reached the maximum value on the 3rd day, higher than that of the CK group, and then the activity decreased. The SOD activity increased in the Y3 and Y7 treatment groups and reached the maximum on the 5th day, which was higher than that of the CK group. The POD activity was significantly increased in the Y1, Y3, and Y7 treatment groups and reached the maximum value on the 5th day, which was higher than that of the CK group, then POD activity decreases. The CAD activity was significantly increased in the Y1, Y3, and Y7 treatment group and reached the maximum value on the 3rd day, higher than that of the CK group, and then the activity decreased. These results suggest that 2,4-di-tert-butylphenol, N-acetyl-5-methoxytryptamine, and P-hydroxybenzoic acid could enhance the disease resistance of rice by inducing an enzymatic defense response.

## 4 Conclusion

In this study, a series of studies were carried out on the fermentation conditions of *P. polymyxa* Y-1, the extraction, isolation, and structural identification of active metabolites, as well as their biological activities. The optimum nitrogen source, carbon source, concentration, temperature, time, and pH value were selected by a single factor experiment. Then eight metabolites were isolated from the fermentation broth of *P. polymyxa* Y-1 by biological activity tracking separation. 2,4-di-tert-butylphenol, N-acetyl-5-methoxytryptamine, P-hydroxybenzoic acid all exhibited good *in vitro* anti-*Xoo* and -*Xoc* activities. The protection experiments of rice plants showed that 2,4-di-tert-butylphenol, N-acetyl-5-methoxytryptamine, and P-hydroxybenzoic acid could protect rice against BLB at 200 μg/ml. Defensive enzyme activity experiments also found that 2,4-di-tert-butylphenol, N-acetyl-5-methoxytryptamine, and P-hydroxybenzoic acid could enhance the disease resistance of rice by inducing an enzymatic defense response. These results will provide important insights into the study of novel microbial pesticides for the treatment of rice bacterial diseases, especially in terms of fermentation conditions, *in vitro* and *in vivo* activities, and mechanisms of action.

## Data Availability

The original contributions presented in the study are included in the article/Supplementary Material, and further inquiries can be directed to the corresponding author.
