# Supplementary material for: Active Metabolites From the Endophyte Paenibacillus polymyxa Y-1 of Dendrobium nobile for the Control of Rice Bacterial Diseases
Source: Front Chem. 2022 Mar 29;10:879724. doi: 10.3389/fchem.2022.879724 (PMC9001896; doi:10.3389/fchem.2022.879724)
Supplement: Supplementary file 1 [file DataSheet1.docx]

Supplementary Material

# **Antibacterial activity assay of *P. polymyxa* Y-1 supernatant**

**
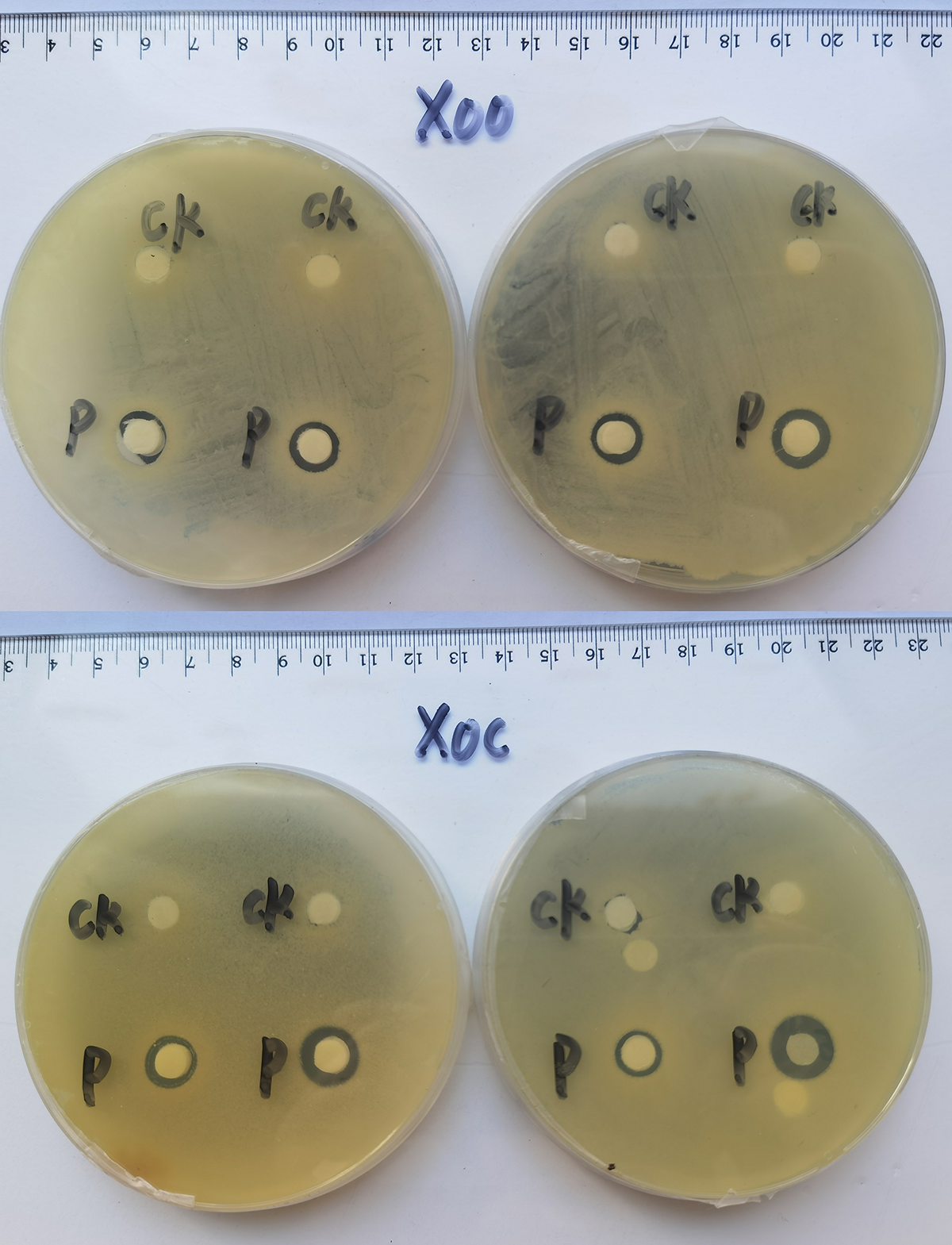
**

**Figure S1** The paper disk method was used to determine the in vitro antibacterial activity of *P. polymyxa* Y-1 supernatant against *Xoo* and *Xoc*.

# **^1^H NMR, ^13^C NMR and HRMS of metabolites Y1-Y10.**


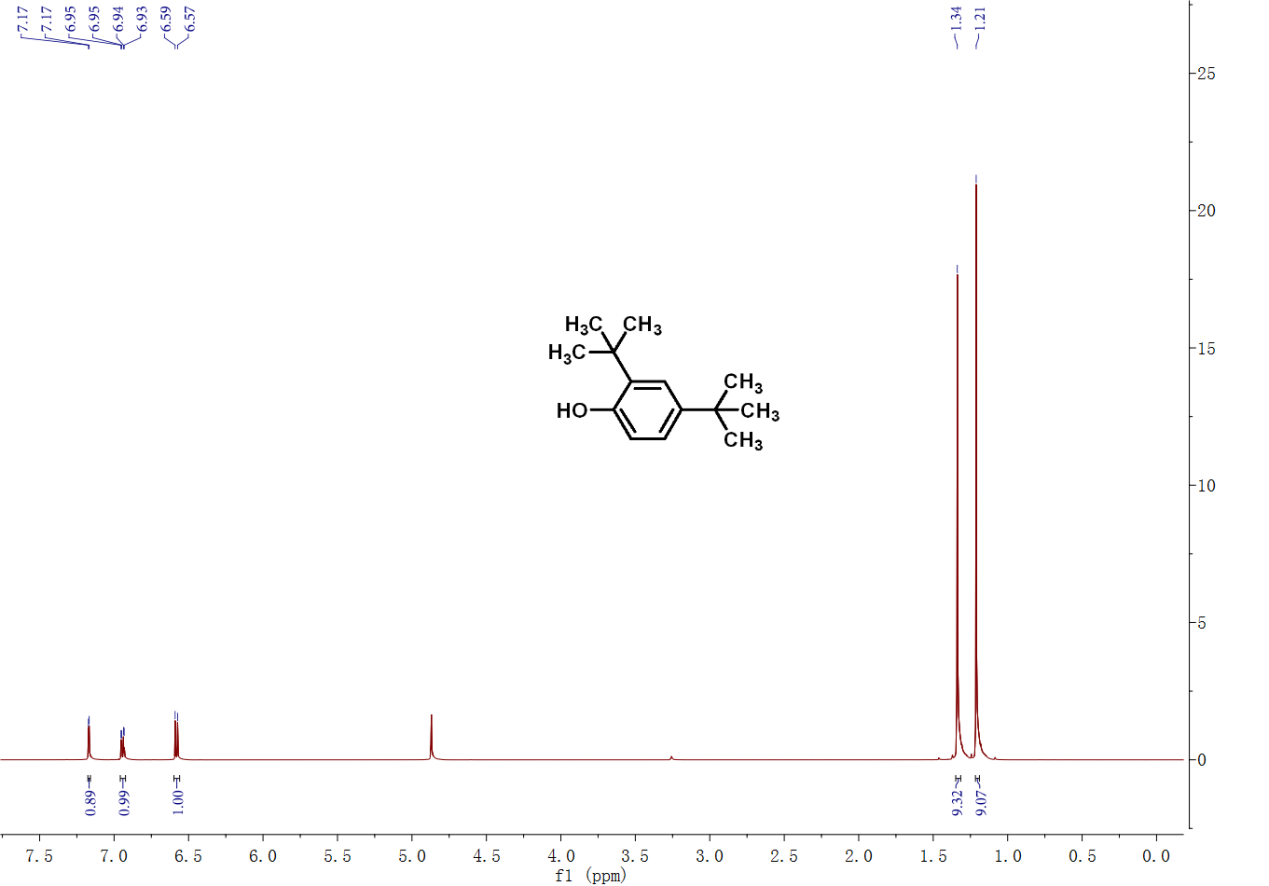


**Figure S2** ^1^H NMR of 2,4-di-tert-butylphenol (Y1)


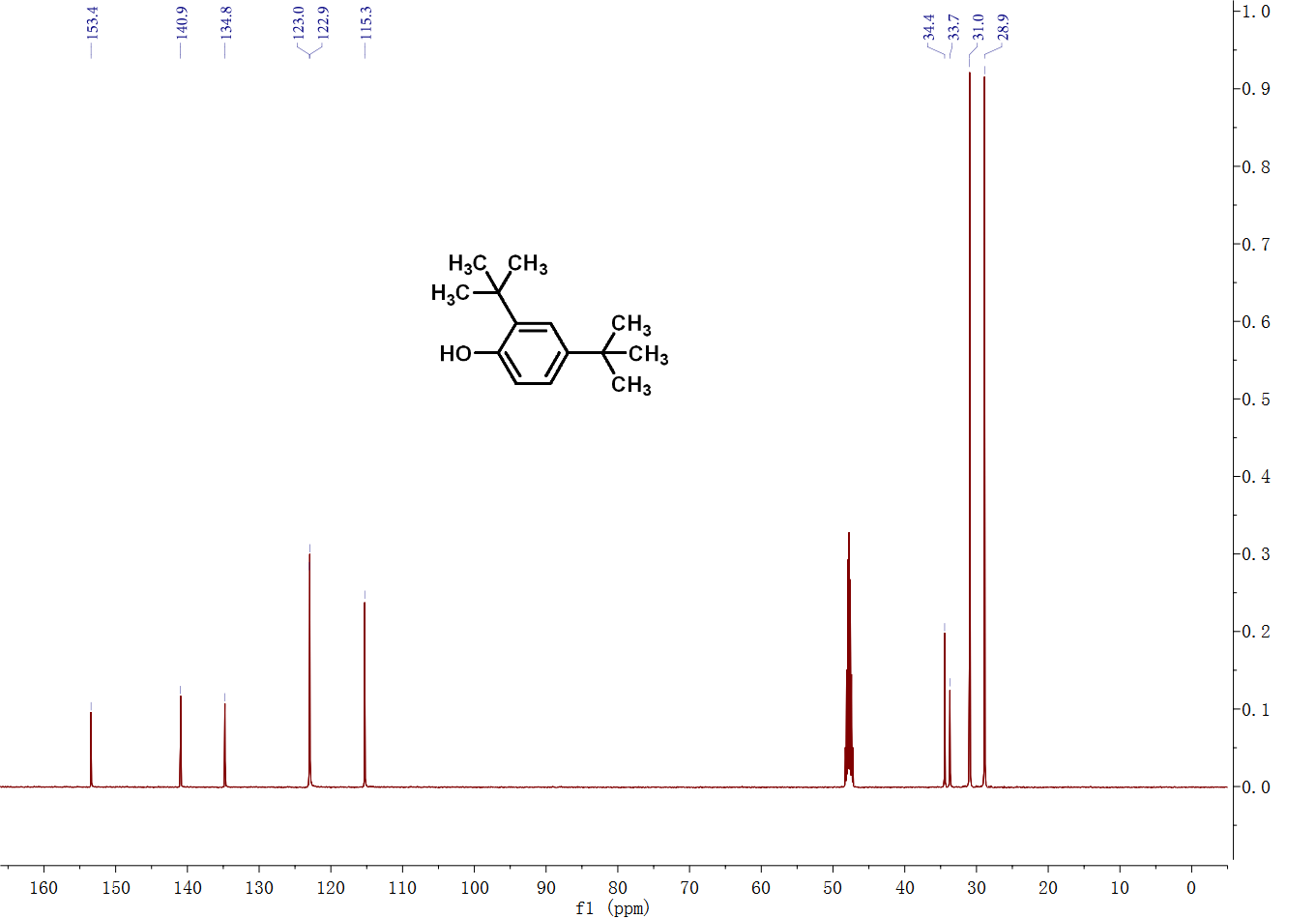


**Figure S3** ^13^C NMR of 2,4-di-tert-butylphenol (Y1)


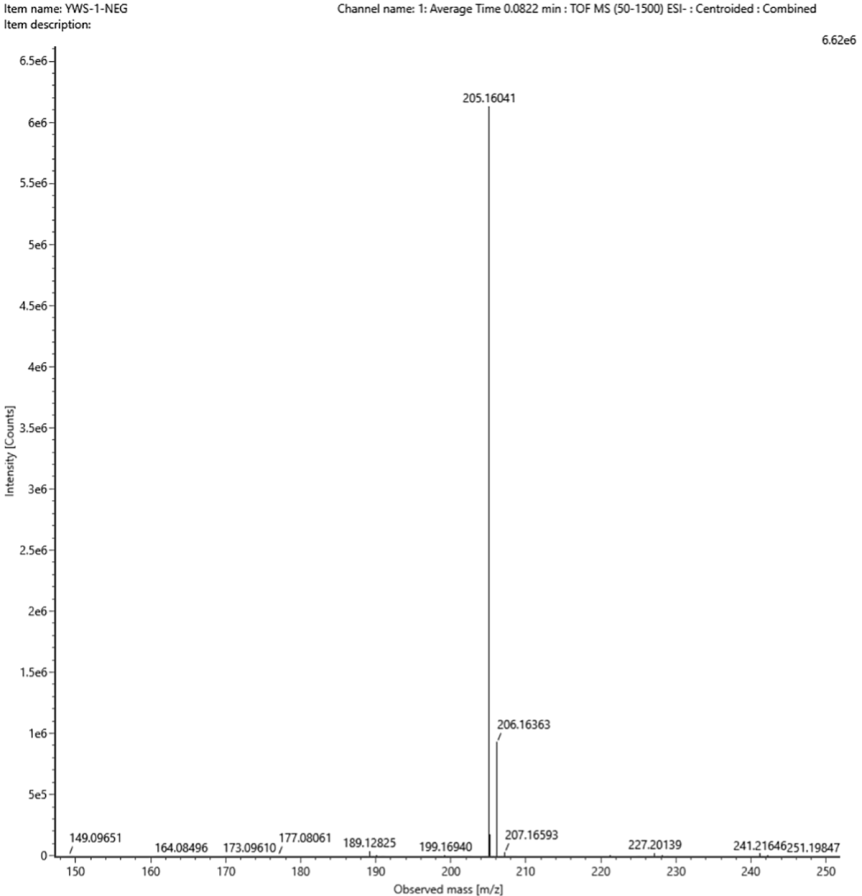


**Figure S4** HRMS of 2,4-di-tert-butylphenol (Y1)


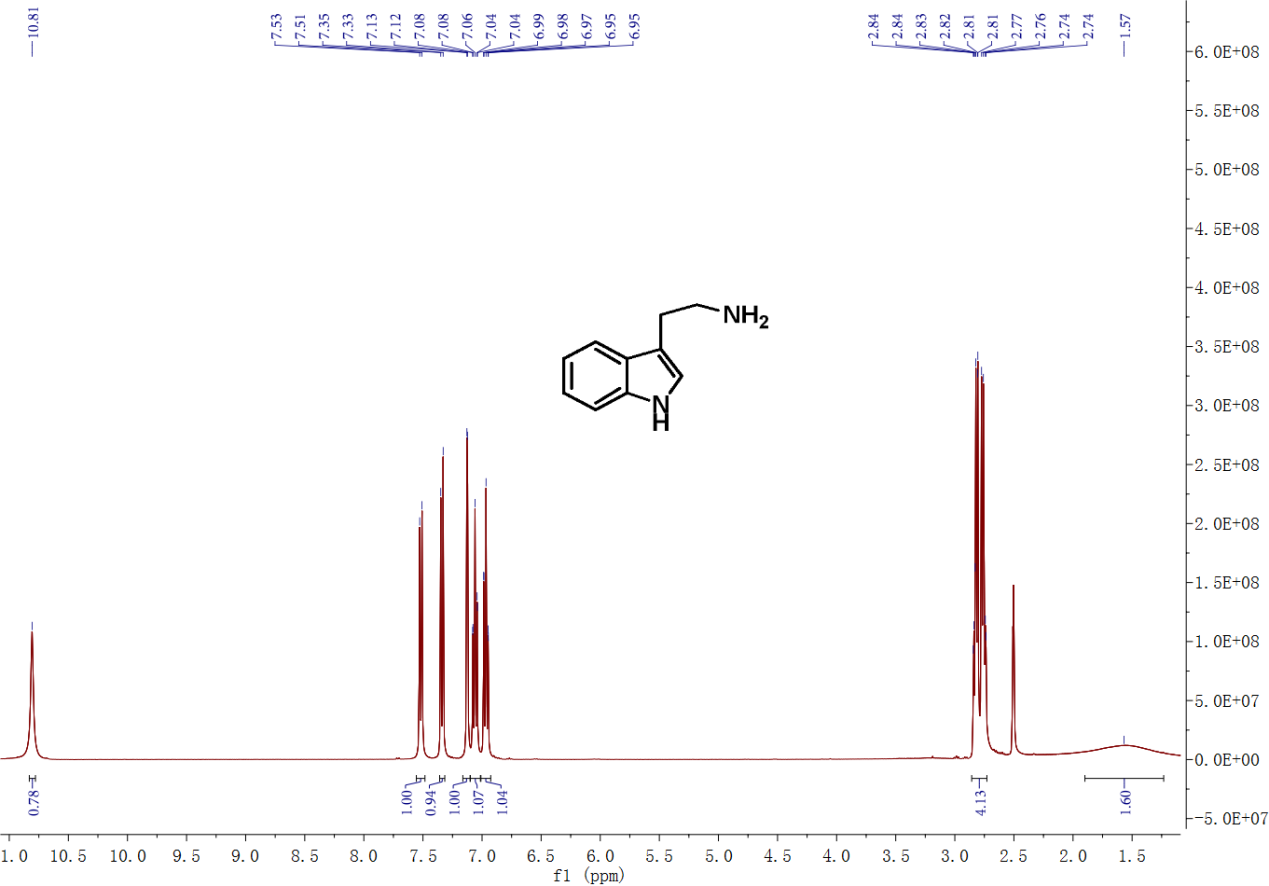


**Figure S5** ^1^H NMR of 3-(2-aminoethyl) indole. (Y2)


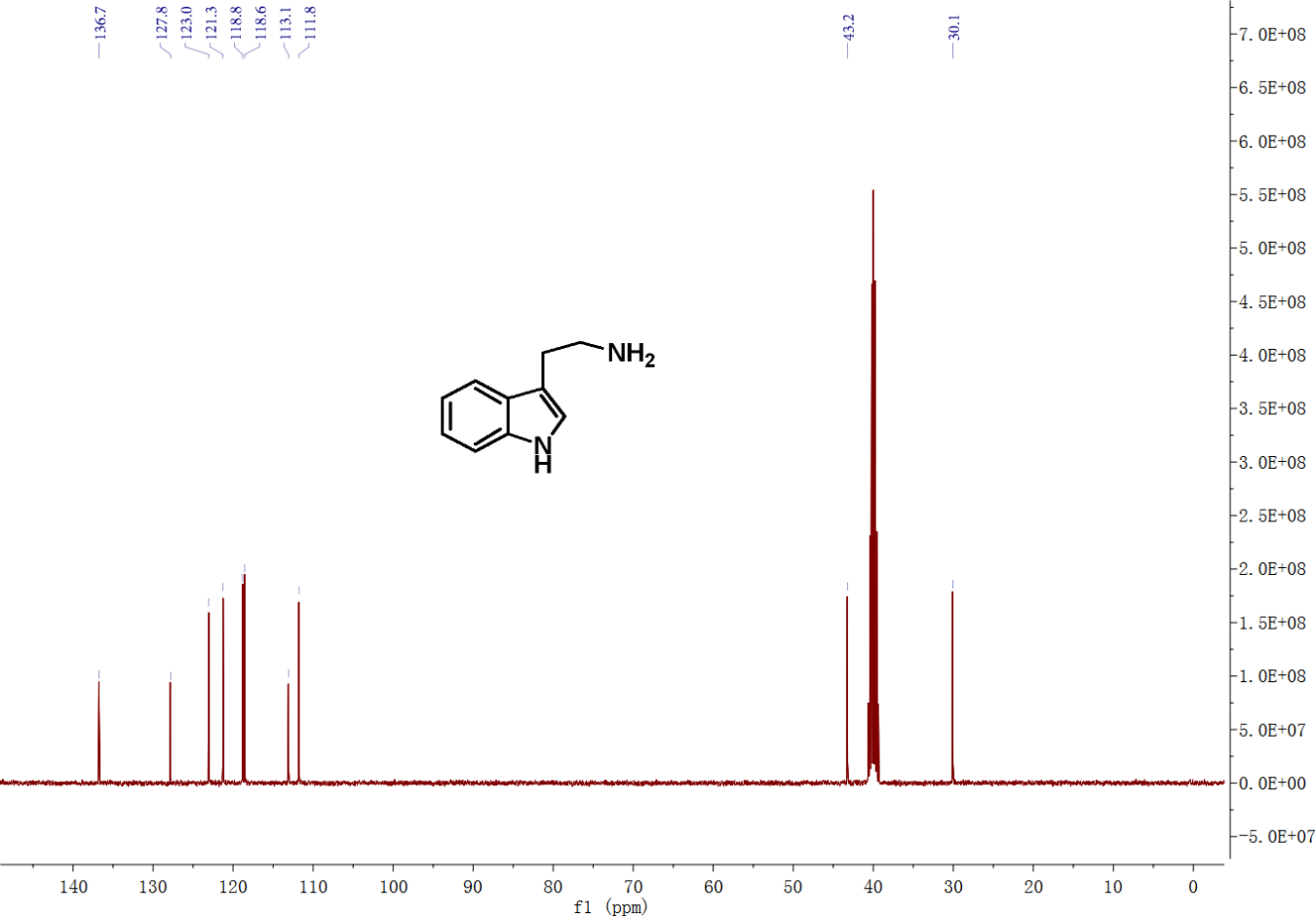


**Figure S6** ^13^C NMR of 3-(2-aminoethyl) indole. (Y2)


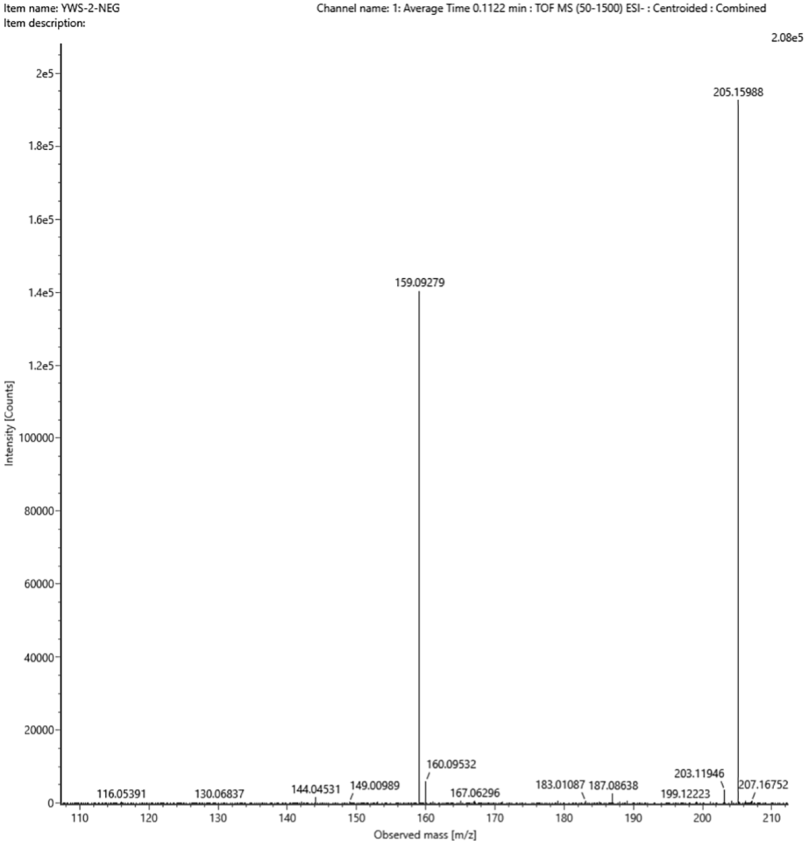


**Figure S7** HRMS of 3-(2-aminoethyl) indole. (Y2)


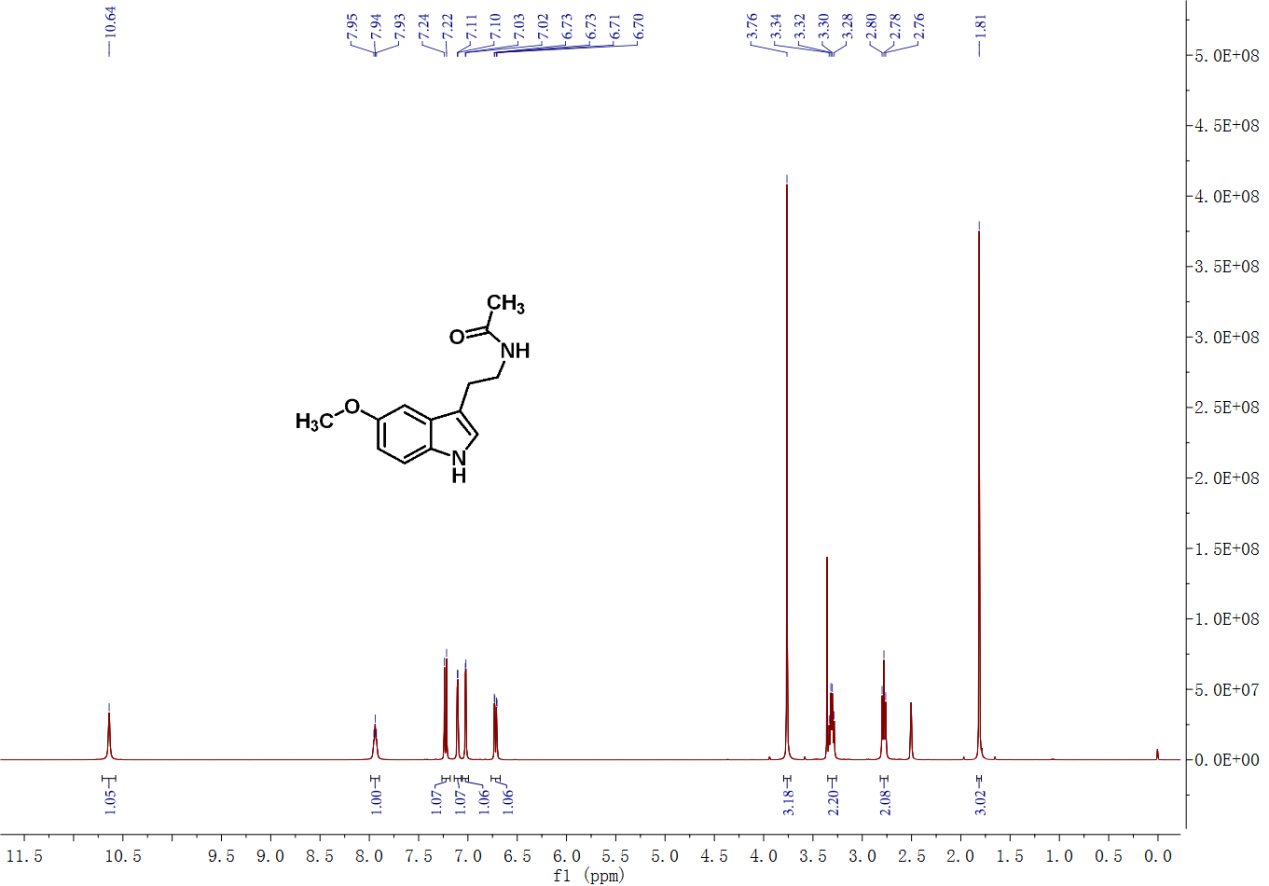


**Figure S8** ^1^H NMR of N-acetyl-5-methoxytryptamine (Y3)


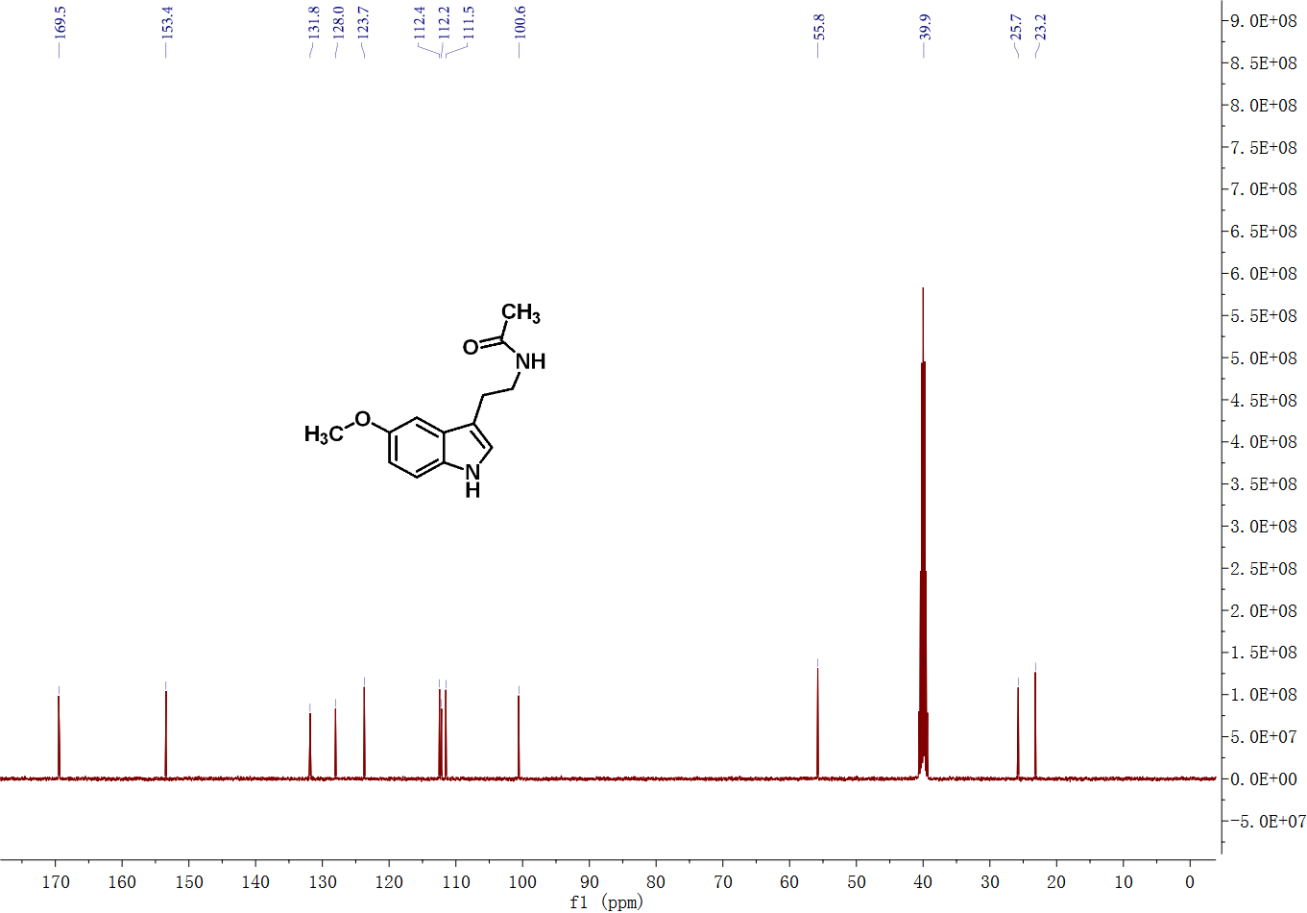


**Figure S9** ^13^C NMR of N-acetyl-5-methoxytryptamine (Y3)


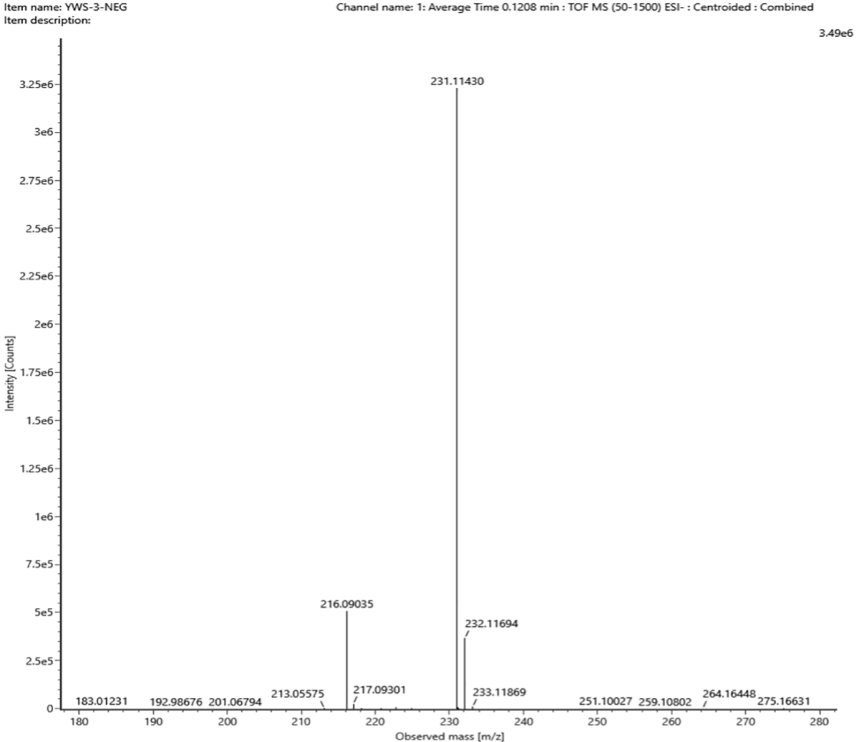


**Figure S10** HRMS of N-acetyl-5-methoxytryptamine (Y3)


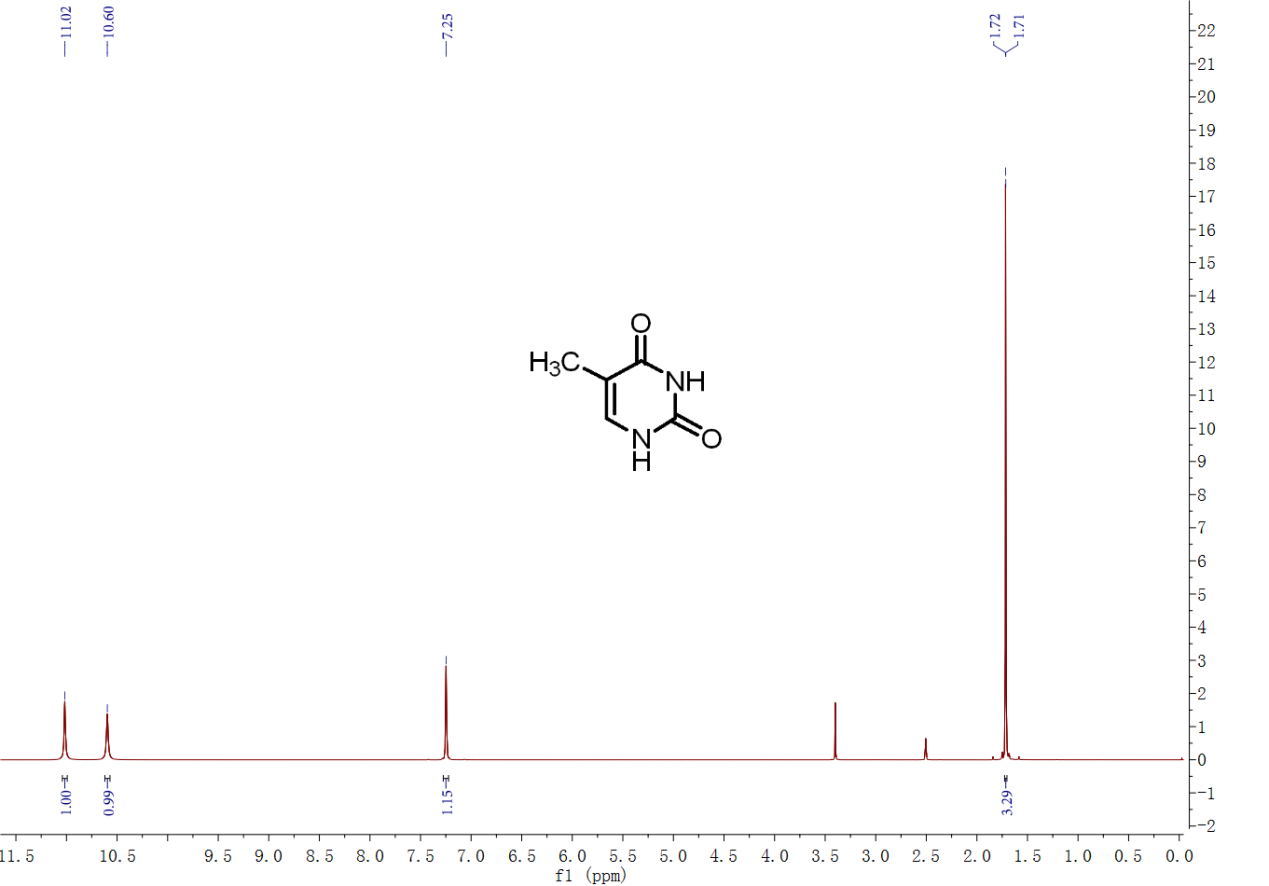


**Figure S11** ^1^H-NMR of 2,4-dihydroxy-5-methylpyrimidine. (Y4)


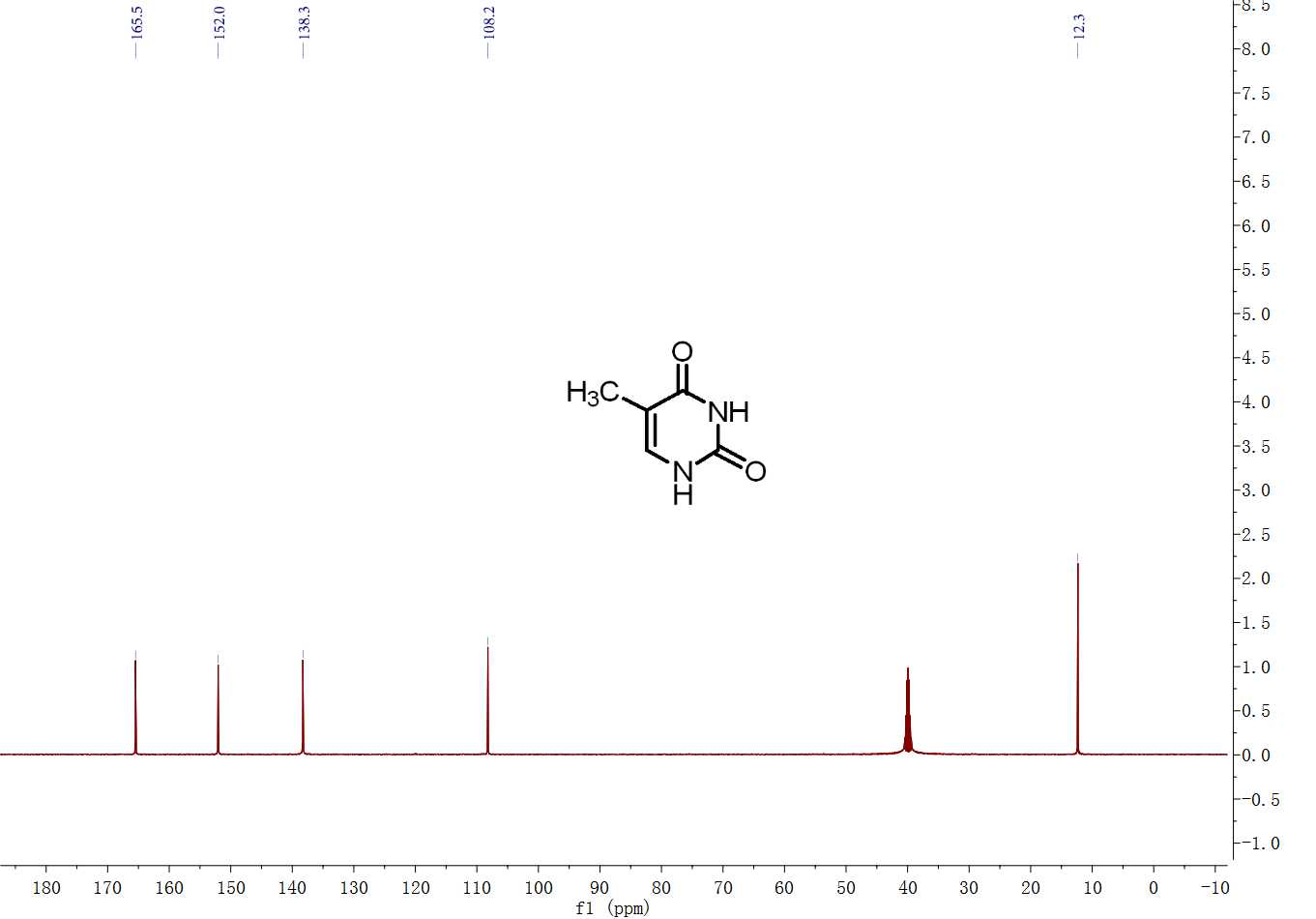


**Figure S12** ^13^C-NMR of 2,4-dihydroxy-5-methylpyrimidine. (Y4)


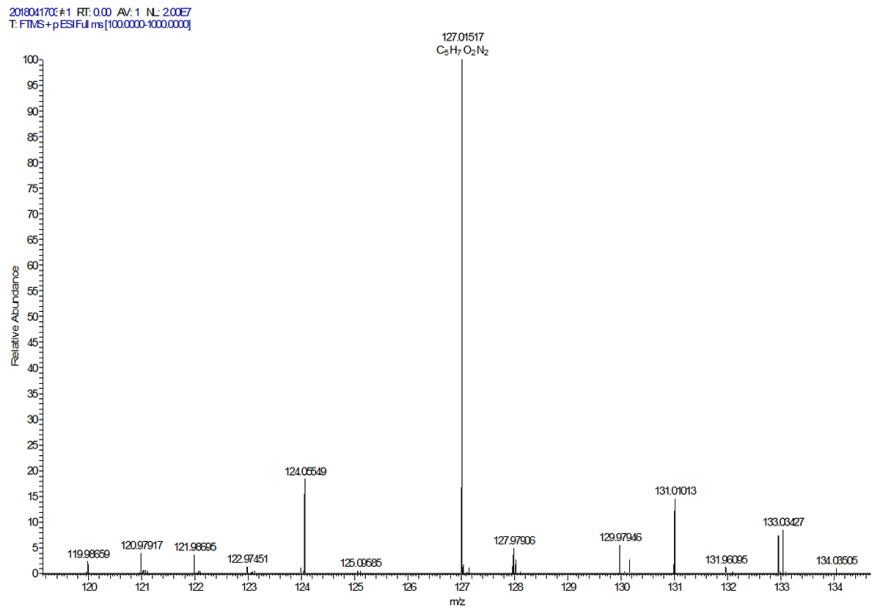


**Figure S13** HRMS of 2,4-dihydroxy-5-methylpyrimidine. (Y4)


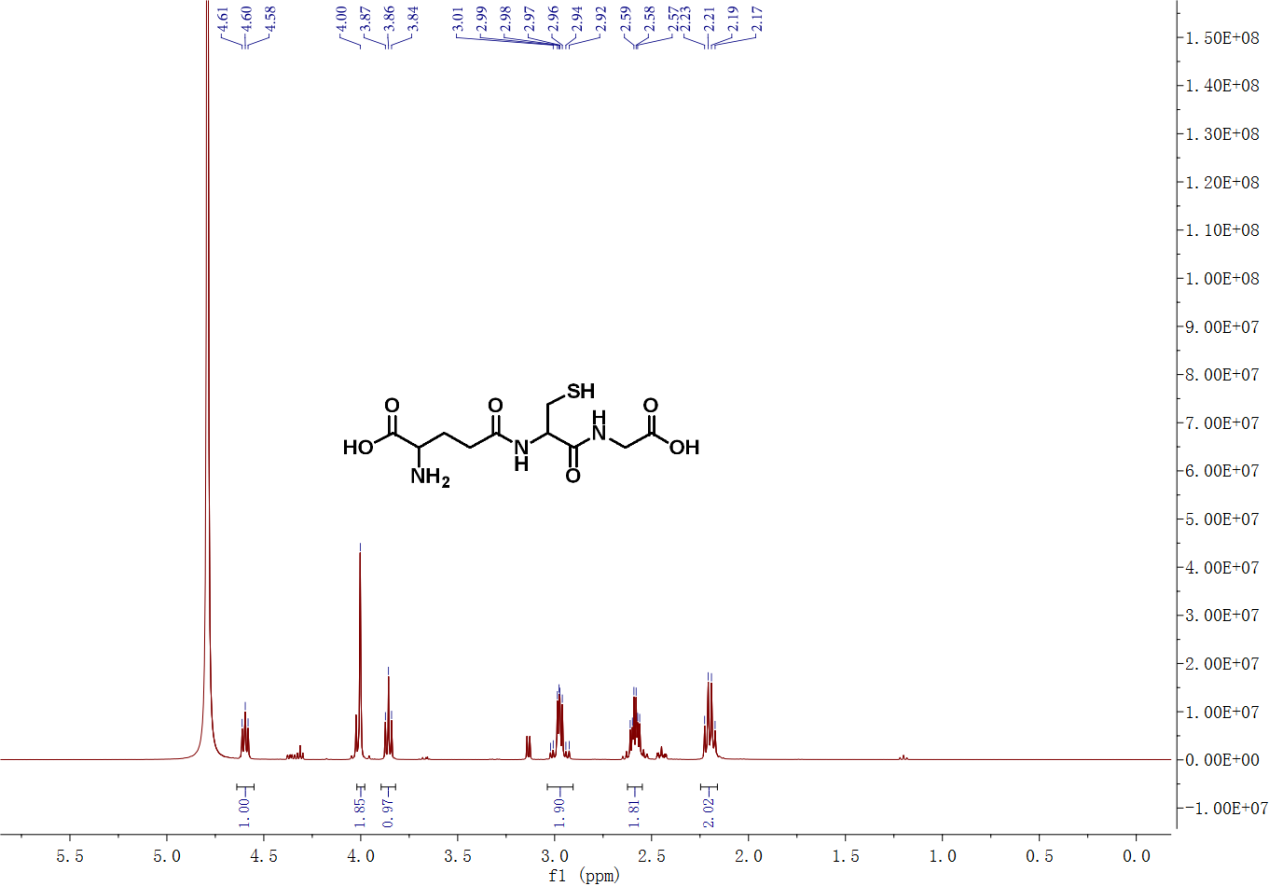


**Figure S14** ^1^H-NMR of glutathione. (Y5)


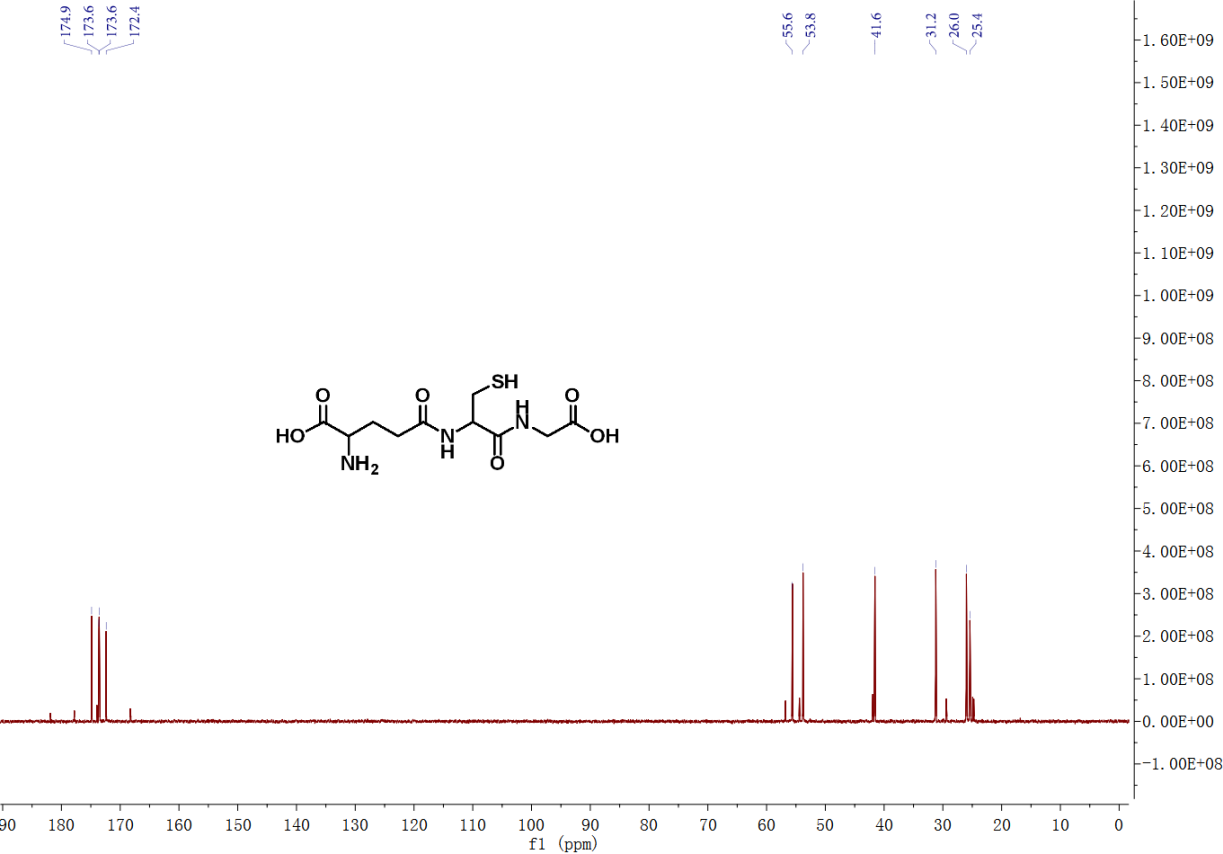


**Figure S15** ^13^C-NMR of glutathione. (Y5)


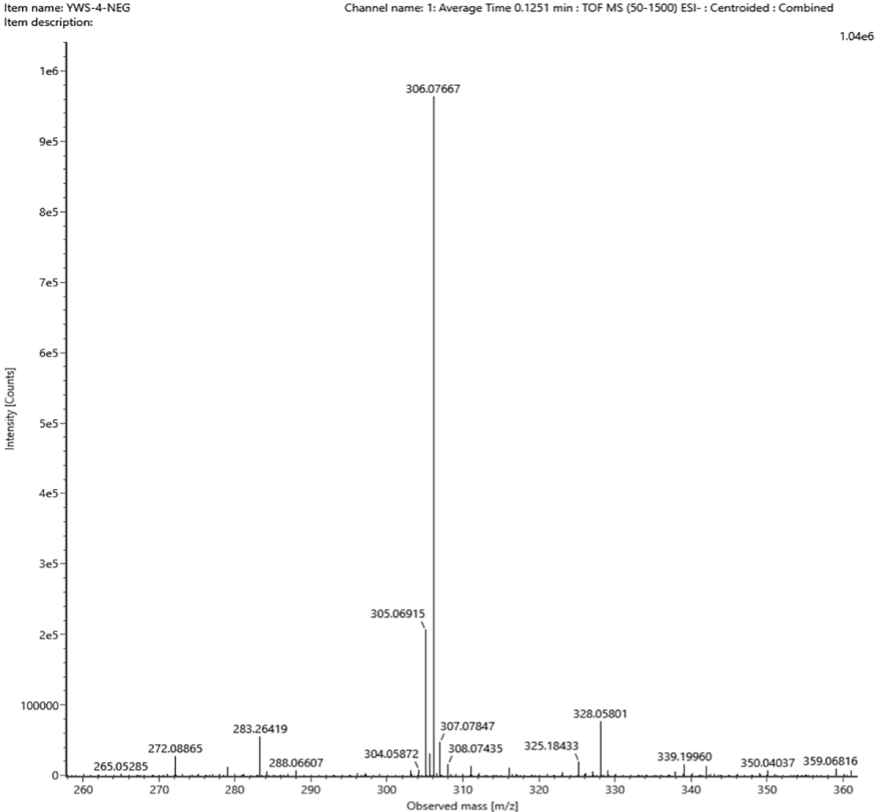


**Figure S16** HRMS of glutathione. (Y5)


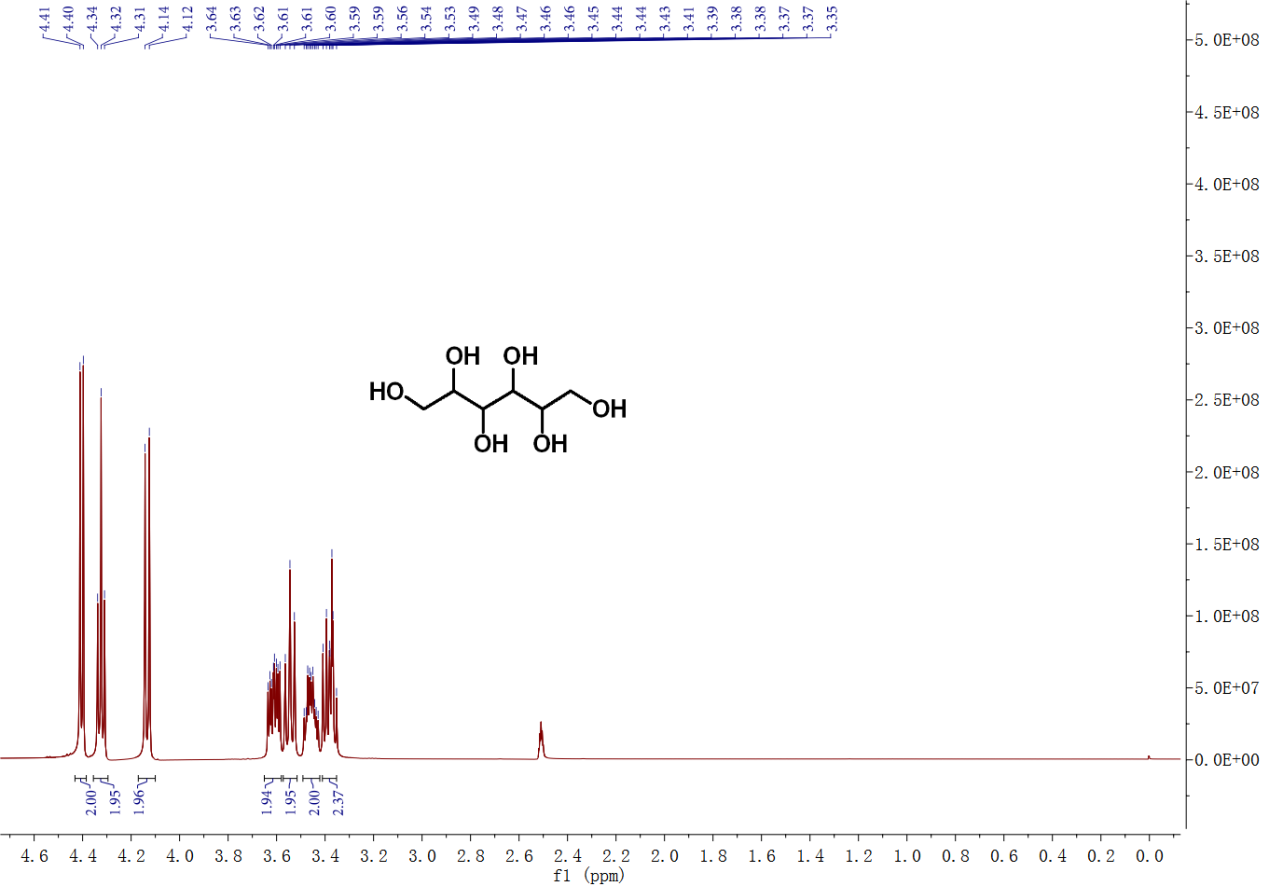


**Figure S17** ^1^H-NMR of mannitol. (Y6)


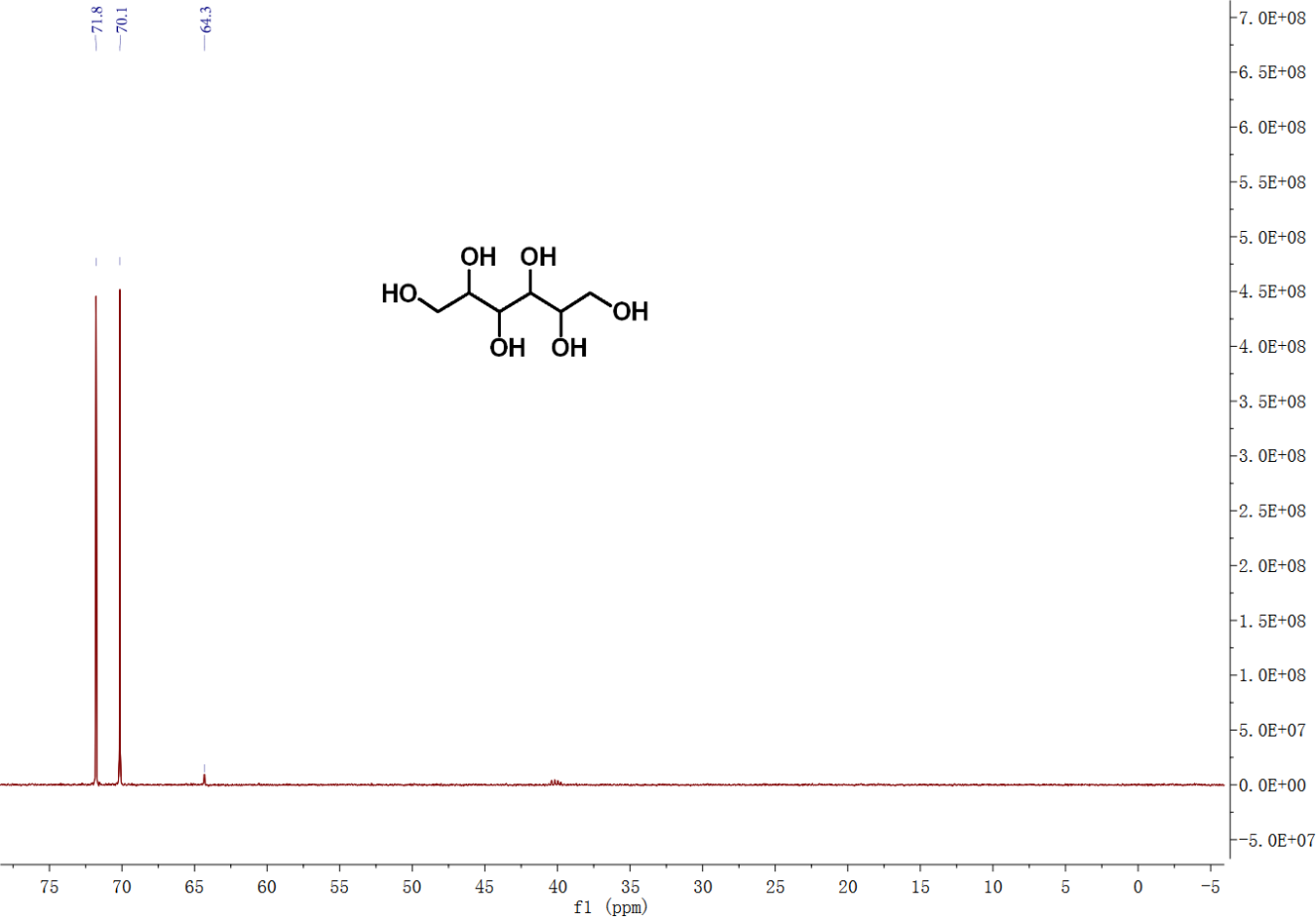


**Figure S18** ^13^C-NMR of mannitol. (Y6)


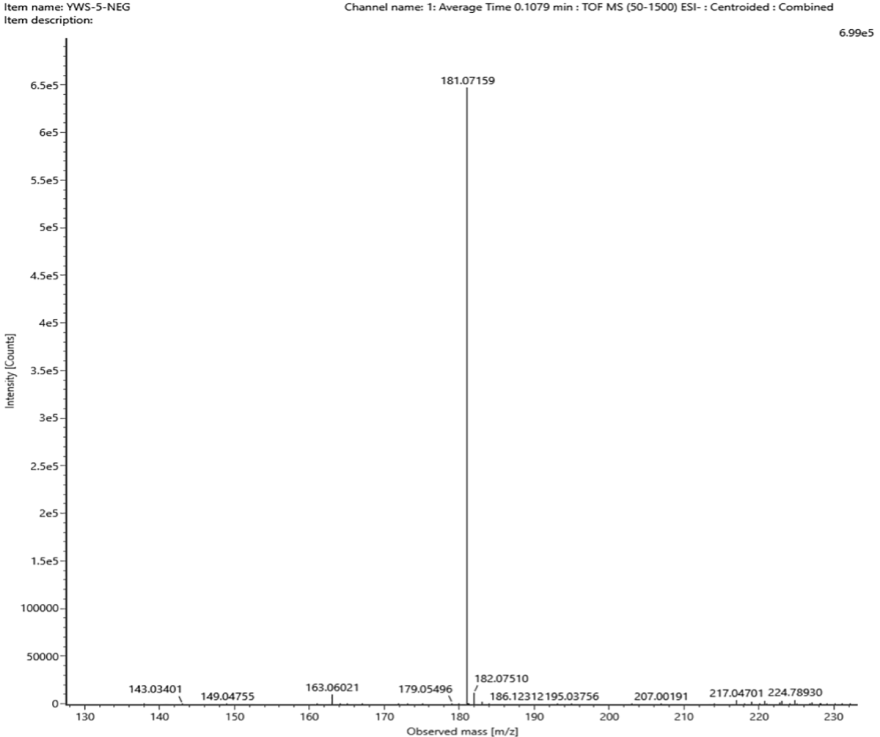


**Figure S19** HRMS of mannitol. (Y6)


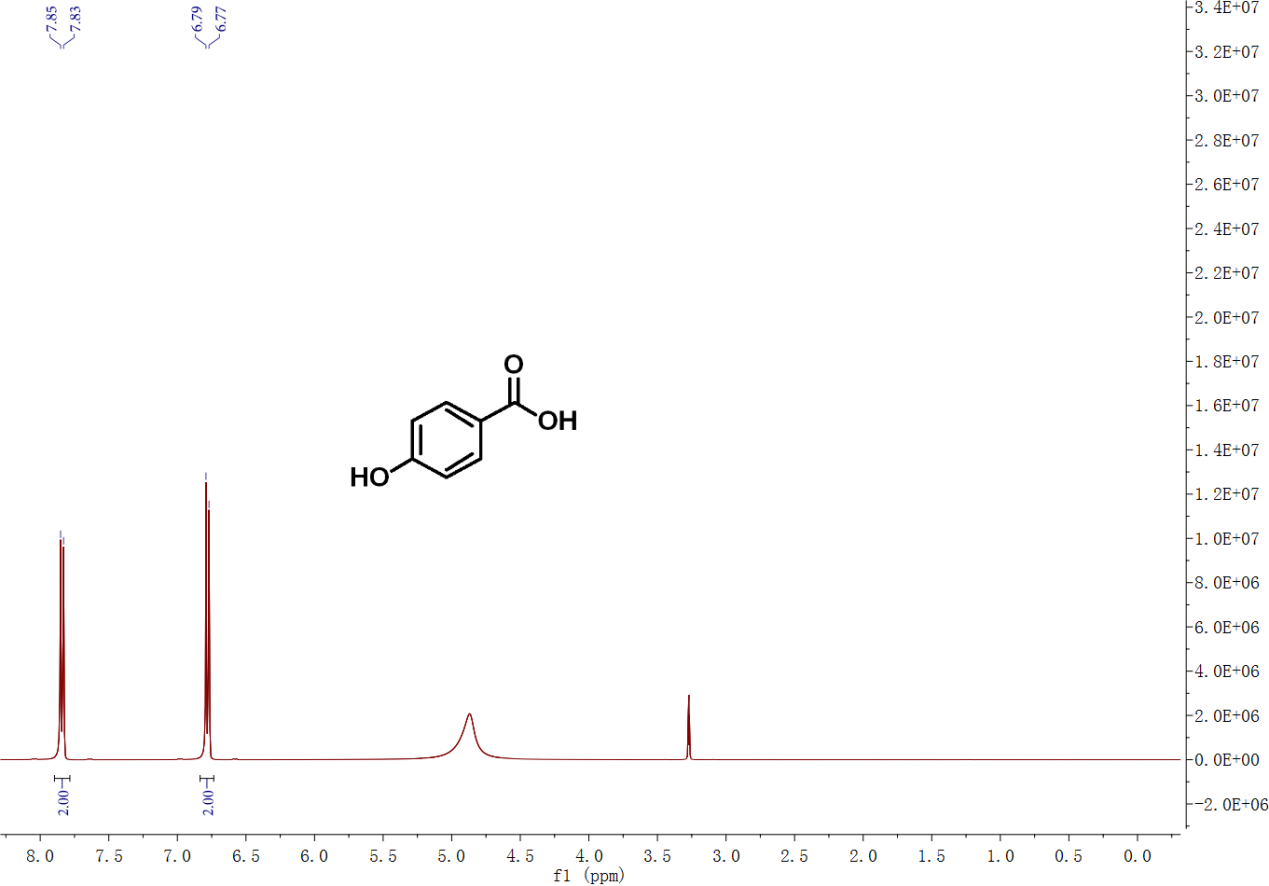


**Figure S20** ^1^H-NMR of P-hydroxybenzoic acid. (Y7)


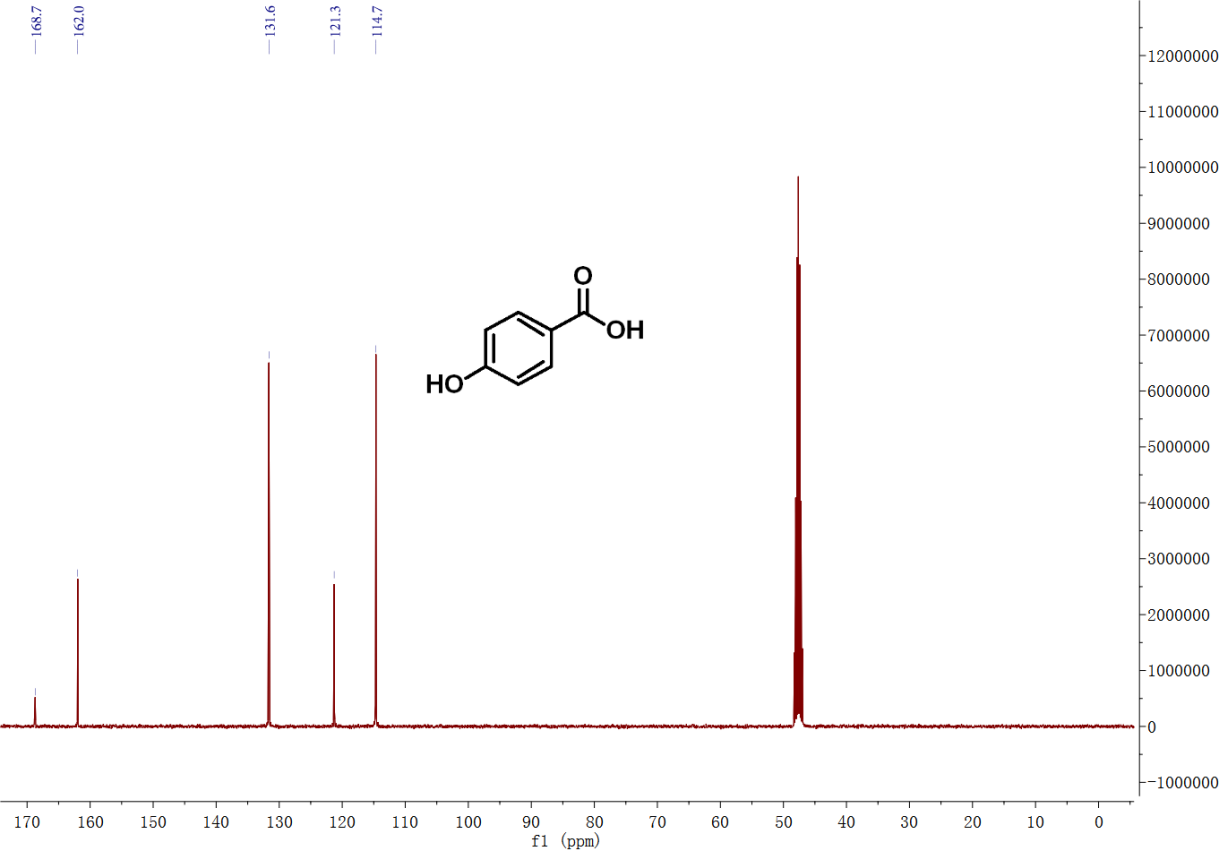


**Figure S21** ^13^C-NMR of P-hydroxybenzoic acid. (Y7)


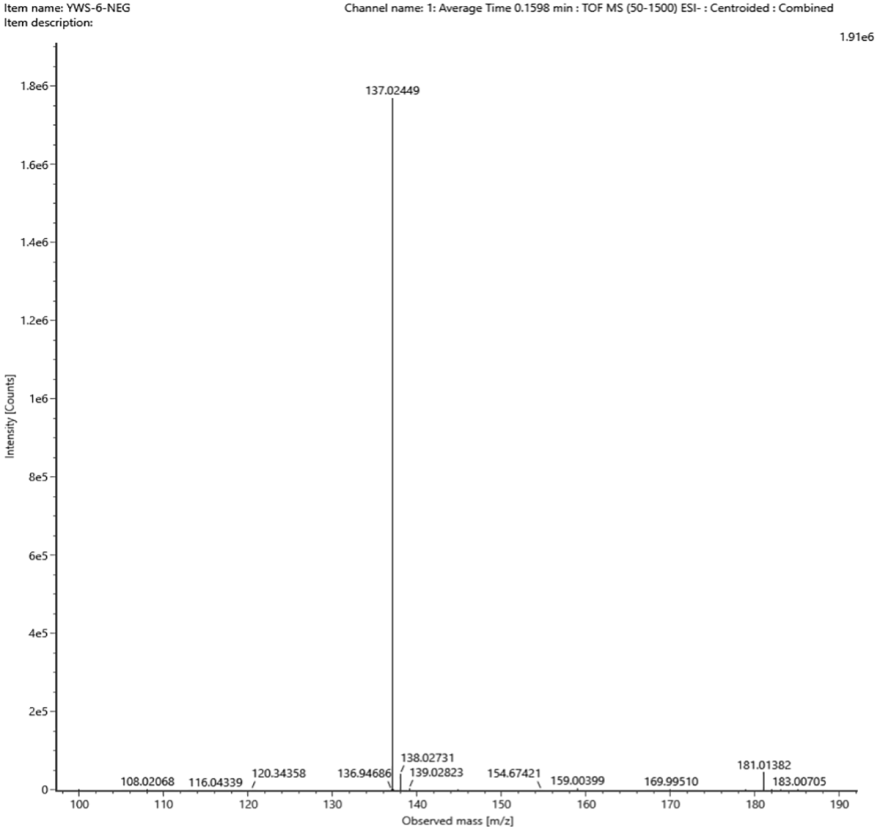


**Figure S22** HRMS of P-hydroxybenzoic acid. (Y7)


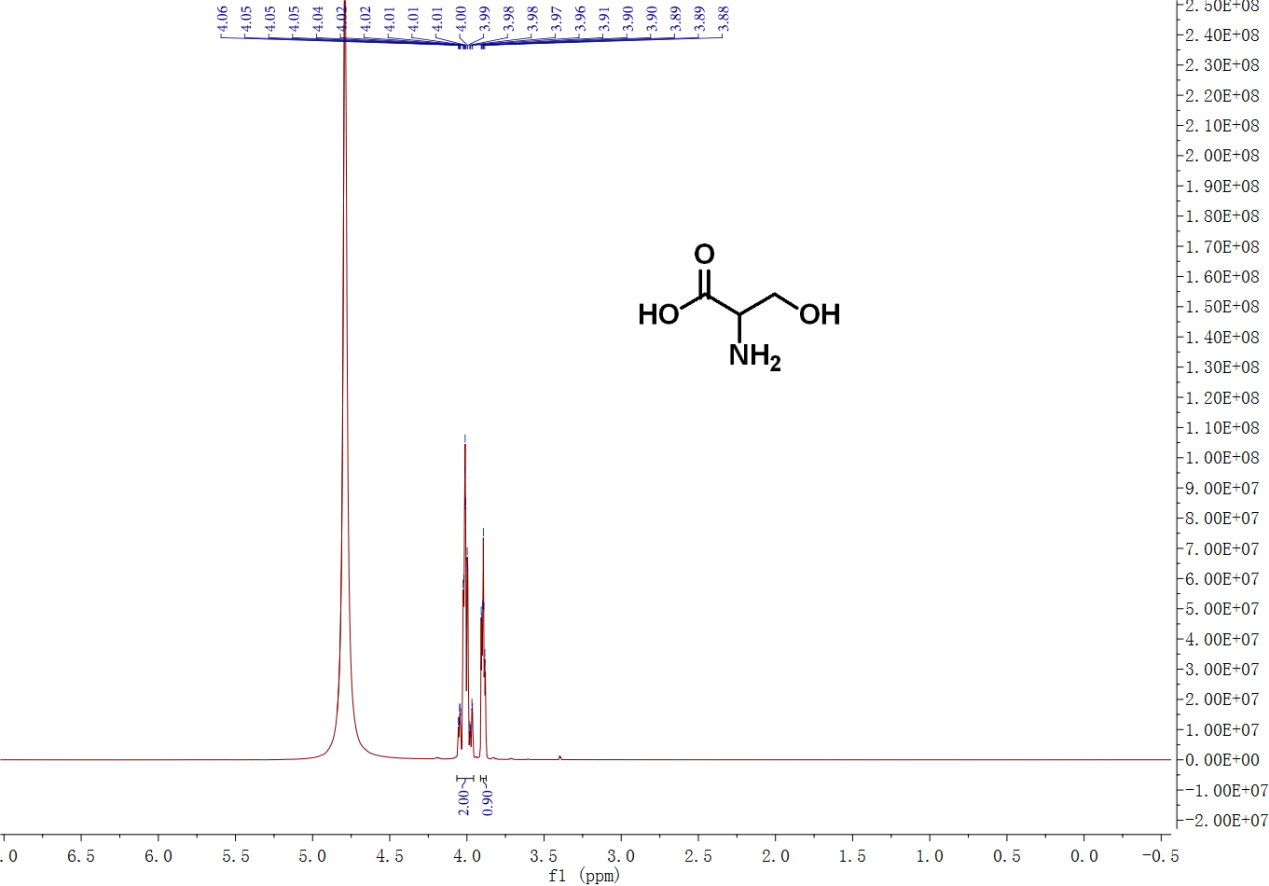


**Figure S23** ^1^H-NMR of serine. (Y8)


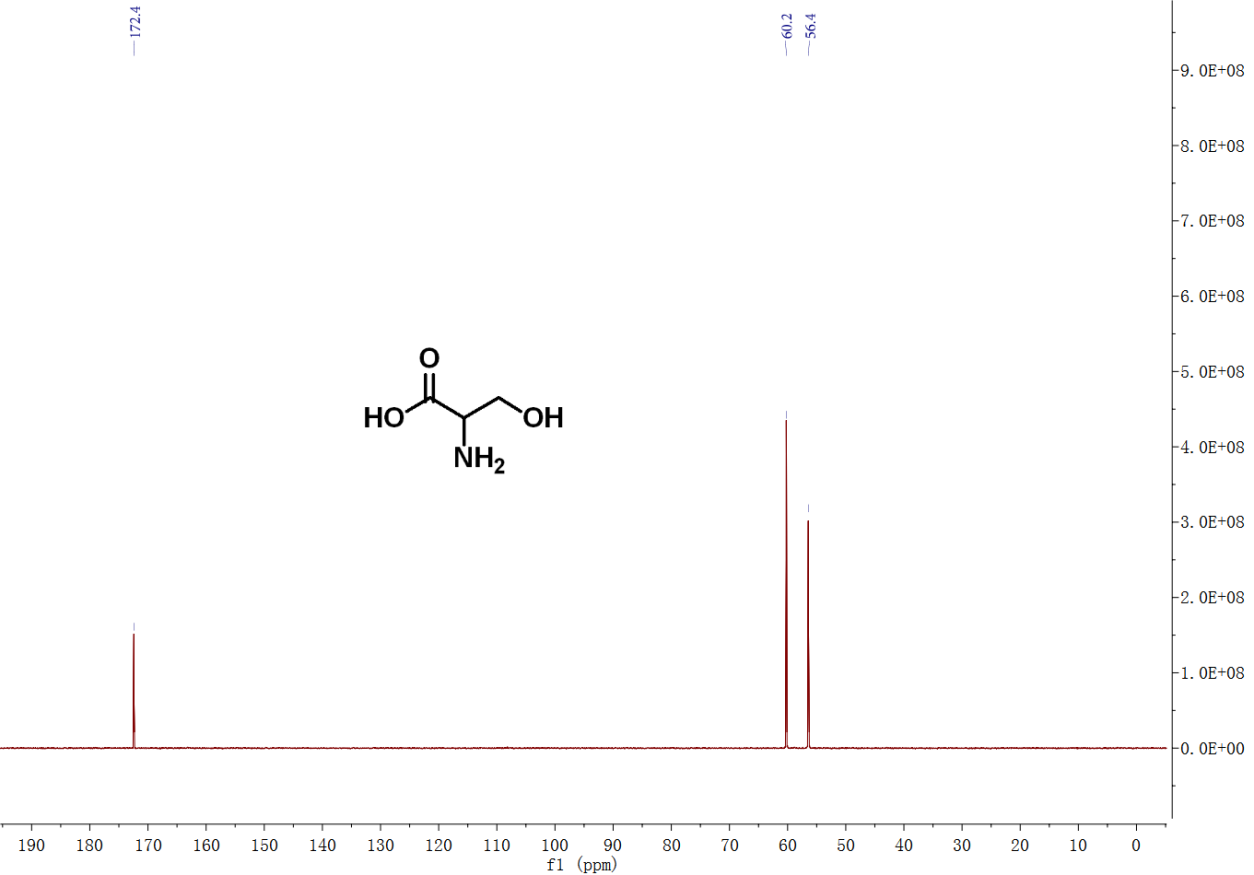


**Figure S24** ^13^C-NMR of serine. (Y8)


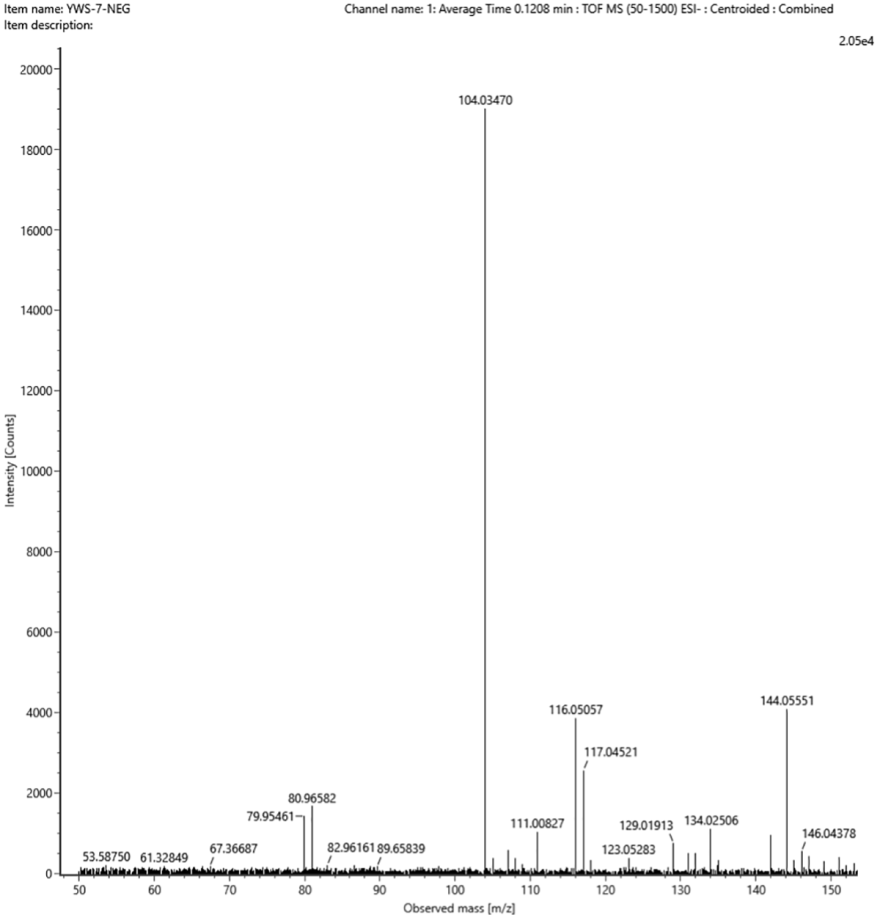


**Figure S25** HRMS of serine. (Y8)
